# Glycosylation Matters: Network Pharmacology-Based and Molecular Docking Analysis of Resveratrol Glycosylated Derivatives on Parkinson’s Disease

**DOI:** 10.1021/acsomega.5c08887

**Published:** 2026-01-23

**Authors:** Lucia E. Schimith, Elvis Martis, Stéphane Teletchea, Ana Luiza Muccillo-Baisch, Corinne André-Miral, Mariana A. Hort

**Affiliations:** † Programa de Pós-graduação em Ciências da Saúde, Faculdade de Medicina, 67820 Universidade Federal do Rio Grande, 96203-900, Rio Grande, RS, Brasil; ‡ 27045 Nantes Université, CNRS, US2B, UMR 6286, 44322, Nantes, France

## Abstract

Resveratrol (RV), a natural polyphenol, has been extensively studied for its neuroprotective potential in Parkinson’s Disease (PD), but its clinical translation is limited by poor bioavailability and rapid metabolism. Glycosylated derivatives, including polydatin and resveratrol-3-α-glucoside, have been proposed to improve the solubility and stability. This study compared the pharmacokinetic properties of RV and its derivatives and examined their molecular interactions with PD-related targets. ADMET (Absorption, Distribution, Metabolism, Excretion, and Toxicity) analysis showed that, despite improved solubility, RV retained a more favorable overall pharmacokinetic profile. Target prediction combined with Gene Ontology (GO) enrichment, Kyoto Encyclopedia of Genes and Genomes (KEGG) pathway analysis, and protein–protein interaction (PPI) network construction identified 50 potential targets, with 11 prioritized for molecular docking. Glycosylated derivatives exhibited binding affinities for all targets stronger than those of RV, with TNF-α, PPARγ, and ERBB2 highlighted as key candidates. These findings indicate that RV glycosylation may enhance therapeutic potential for PD treatment by promoting stronger molecular interactions with critical targets, though *in vivo* validation remains necessary.

## Graphical abstract

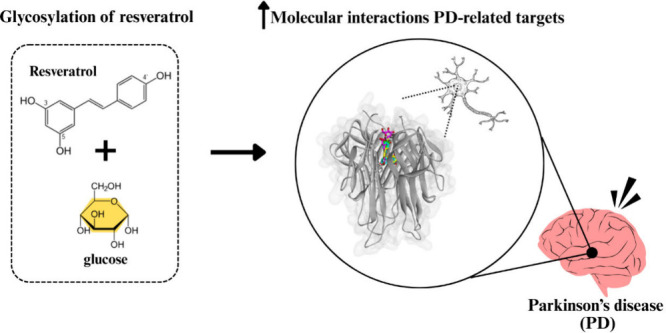

## 1 Introduction

Parkinson’s disease (PD) is a progressive neurodegenerative disorder characterized by the loss of dopaminergic neurons in the substantia nigra, leading to motor symptoms such as bradykinesia, tremor, rigidity, and postural instability, as well as nonmotor complications, including neuropsychiatric and sensory disturbances, cognitive decline, autonomic dysfunction, sleep disorders, and pain.^1,2^ The World Health Organization estimates that the prevalence of PD has doubled over the past few decades. As the disease progresses, clinical symptoms and complications significantly affect patients’ quality of life, resulting in disability and an increased need for long-term care.^3,4^

While several approved pharmacological therapies effectively alleviate motor symptoms and improve the mobility of PD patients, no available therapy currently slows disease progression or promotes relief for the wide spectrum of clinical manifestations. The multifactorial nature of PD makes the development of new potential drugs even more complicated, considering the multiple pathological mechanisms, involving α-synuclein aggregation, oxidative stress, mitochondrial dysfunction, and neuroinflammation, as well as heterogeneous disease progression.^5,6^

Natural compounds are being explored as a potential source of therapeutic alternatives for PD due to their multitarget characteristics. While currently available conventional treatments focus on controlling symptoms, natural molecules have demonstrated promise in managing the underlying neurodegenerative processes mainly as a result of their different neuroprotective properties.^7,8^ Resveratrol (3,4′,5-*trans*-trihydroxystilbene) (RV) is a naturally occurring polyphenol widely recognized for its biological activities such as antioxidant, anti-inflammatory, and antiapoptotic, showing potential therapeutic effects in experimental models of PD.^9–11^

Exploring natural molecules for therapeutic use encounters difficulties, particularly related to pharmacokinetics and bioavailability. The development of glycosylated analogues via biosynthetic pathways can enhance pharmacological properties, potentially improving pharmacokinetics while reducing toxicity and increasing target-specific activity.^12,13^ Polydatin (resveratrol-3-*O*-β-d-glucoside) and resveratrol-3-α-glucoside (resveratrol-3-*O*-α-d-glucoside) are glycosylated derivatives of RV that have demonstrated improvements in stability and solubility compared to RV, leading to better absorption and prolonged systemic circulation^14^ (Figure 1). Polydatin, a natural precursor of RV, was originally isolated from *Polygonum cuspidatum*,^15^ whereas resveratrol-3-α-glucoside was synthesized via enzymatic glycosylation using sucrose phosphorylases from *Bifidobacterium adolescentis* (BaSP) with Q345F mutation, which enlarges the active site entry to accommodate polyphenols into the catalytic pocket of BaSP.^16,17^ While biological activities of polydatin largely overlap with those of RV, its glycosylated precursor has been investigated for its antioxidant^18,19^ and anti-inflammatory^20^ properties that may surpass those of its aglycone counterpart.^21,22^ Although *in vitro* or *in vivo* studies have not been conducted on the biological activities of resveratrol-3-α-glucoside, Akash and colleagues^23^ recently evaluated its anticolorectal cancer potential through *in silico* methods.

**Figure 1:**
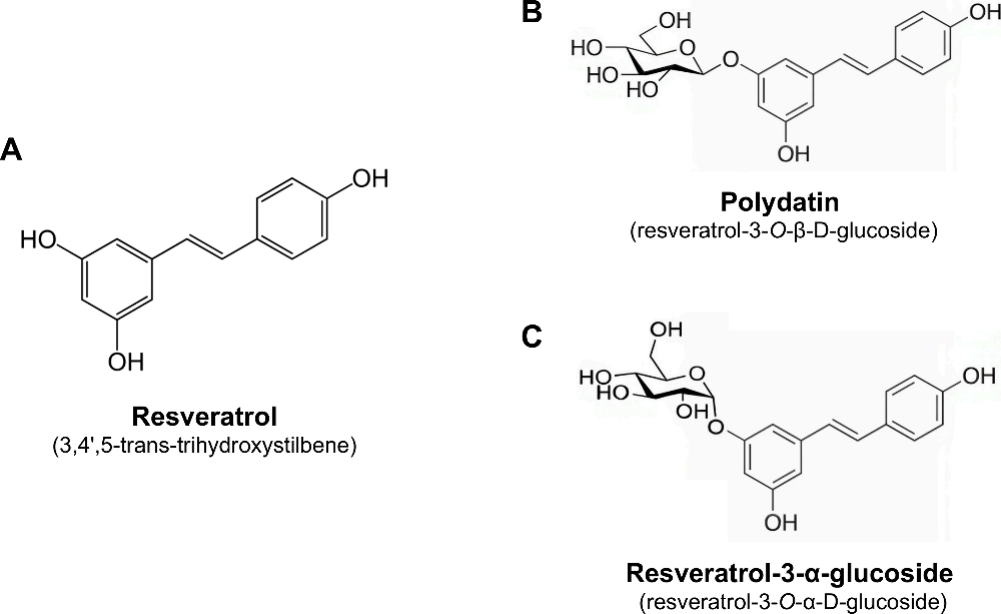
Chemical structure of (A) resveratrol, (B) polydatin, and (C) resveratrol-3-α-glucoside.

*In silico* approaches have become powerful tools in drug discovery and development processes, offering several advantages by increasing cost effectiveness, reducing time investment, and minimizing animal testing, especially on initial analyses.^24^ Network pharmacology tools provide innovative insights into drug candidate identification, operating in opposite to the traditional “one drug, one target” models and allowing a more comprehensive approach based on modulation of multiple targets in disease progression and potential therapeutic effects.^25^ Using computational analyses, we assessed the impact of RV glycosylation and the glycosidic bond type on the pharmacological interactions of the glycosylated derivatives polydatin and resveratrol-3-α-glucoside with key molecular targets associated with PD by comparing their binding affinities and potential neuroprotective effects.

## 2 Materials and Methods

### 2.1 Prediction of Pharmacokinetic and Pharmacodynamic Profiles

The absorption, distribution, metabolism and excretion (ADME) and toxicity parameters of RV, polydatin, and resveratrol-3-α-glucoside were predicted using the free online platforms ADMETLab 3.0 (https://admetlab3.scbdd.com/) and ProTox 3.0 (https://tox.charite.de/). Potential molecular targets of the three compounds were identified through searches on the SwissTargetPrediction (http://www.swisstargetprediction.ch/) and Comparative Toxicogenomics Database (CTD) (https://ctdbase.org/). Results were acquired by importing the SMILES (simplified molecular-input line-entry system) code of the compounds into the platforms. The resulting three target data sets were merged, and duplicate entries were removed.

### 2.2 Protein–Protein Interaction (PPI) Network

Targets related to PD were retrieved from The Human Gene Database (GeneCards) (https://www.genecards.org/) using the keyword “Parkinson’s disease”. The common predicted targets of RV, polydatin and resveratrol-3-α-glucoside were compared with the PD-related targets and the final cluster of genes was then imported into the STRING platform (https://string-db.org/) to construct a PPI network. The screening analysis conditions were set to *Homo sapiens* as the organism, with a confidence score of 0.4, and all active interaction sources selected. The resulting data from STRING were imported into Cytoscape 3.8.2 software where the PPI network was built and analyzed using the “Network Analyzer” tool. Degree value, betweenness centrality, and closeness centrality values were computed for all nodes. Targets showing degree ≥ 13, betweenness centrality ≥ 0.03, and closeness centrality ≥ 0.4 were selected, following the approach described by Sun et al. (2023).^26^ These thresholds highlight the most influential nodes within the PPI network. Degree value refers to the number of direct connections a node has with other nodes in the network; the higher the degree value, the more essential the protein is for the biological interaction. Betweenness centrality is related to the frequency with which the node appears on the shortest connection between two other nodes. Elevated betweenness centrality values indicate a protein that plays a key role in facilitating network connections. Closeness centrality measures the distance of a node to all of the other nodes in the network, calculating the average of the shortest distance to each of them. This distance can be directly related to the speed of signal transmission between nodes.^27^ The network diagram illustrating the interactions between the three polyphenol candidates and PD was obtained, with the color intensity of the nodes representing degree values, while the edge thickness indicated combined score values.

### 2.3 Gene Ontology Enrichment and KEGG Pathways Analyses

To identify the biological functions, molecular interactions and biochemical pathways of the potential targets of RV and its glycosylated forms in relation to the pathology of PD, gene ontology (GO) enrichment and Kyoto Encyclopedia of Genes and Genomes (KEGG) pathways analyses were conducted using DAVID database (https://davidbioinformatics.nih.gov/). Species were set as *Homo sapiens* and the cutoff value set as *p* ≤ 0.05.^28^ Through GO analysis, it is possible to identify the biological process (BP), cell component (CC), and molecular function (MF) of genes involved in the network. KEGG pathway analysis focuses on identifying the complex networks of molecular interactions and biochemical processes, connecting genes and proteins within the context of a biological system as the disease development and progression.^29,30^ By analyzing these pathways, we can show how specific proteins or gene products contribute to the development of pathology and identify key molecular targets and signaling networks that may be modulated with a view to therapeutic interventions. The top 15 genes of GO and the top 20 pathways of KEGG analyses with significant p value were selected to draw the diagrams using SRplot platform (https://www.bioinformatics.com.cn/en).

### 2.4 Molecular Docking

The 3D structures of RV and polydatin were downloaded from PubChem (https://pubchem.ncbi.nlm.nih.gov/), while the structure of resveratrol-3-α-glucoside was manually drawn using Maestro-GUI software by Schrödinger, Inc. (https://www.schrodinger.com/platform/products/maestro/). Receptor structures were selected from Protein Data Bank (PDB) (http://www.rcsb.org/pdb/) and downloaded in PDB format. Ligand and receptor files were prepared using UCSF Chimera v1.18 software (https://www.cgl.ucsf.edu/chimera/). Standard processing steps included water removal, structural minimization, addition of hydrogen atoms, assignment of Amber ff14SB force field for standard residues, and Gasteiger charges for nonstandard residues. For cases where protein structures required corrections (e.g., missing or broken chain regions), Chimera’s loop modeling and structure refinement tools were applied. Docking parameters were defined as follows: 1) the centroid of each cocrystallized ligand was used to define the grid center coordinates (*x*/*y*/*z*), in order to define the location of the receptor’s active site; 2) grid box size was set as 20 × 20 × 20 Å^3^ (*x*/*y*/*z*) to ensure complete ligand coverage; 3) advanced docking parameters included number of binding modes set as 9, exhaustiveness level of 8, and a maximum energy difference of 3 kcal/mol. PDB ID codes, grid box coordinates, and dimensions can be found in Table S1 in the Supporting Information. Molecular docking calculations were performed using executable file of AutoDock Vina v1.2.x (https://vina.scripps.edu/) integrated into Chimera software. For docking validation, the cocrystallized ligands from each PDB complex structure were redocked into their respective binding sites using the same parameters applied for RV and glycosylated derivatives. The resulting docked poses were compared to the experimentally determined conformations to ensure the accuracy of the docking procedure. Results were expressed as the binding affinity (kcal/mol). The resulting PDBQT files were converted to PDB format using PyMOL (https://www.pymol.org/), and receptor–ligand complexes from the first poses were uploaded to Discovery Studio (BIOVIA) (https://www.3ds.com/products/biovia/discovery-studio/visualization) for visualization and interactions analyses.


**PDB ID Codes**
 TNF
7JRA
EGFR
1M17
ALB
1H9Z
CASP3
1NMS
ESR1
1XPC
PPARγ
1FM6
PTGS2
5IKR
ERBB2
3RCD
SRC
8JN9
ACE
1UZE
MAP2K1
3VVH


## 3 Results

### 3.1 Predicted Physicochemical, Pharmacokinetic, and Toxicological Profiles of RV and Its Glycosylated Derivatives

The predicted physicochemical and ADME properties of RV, polydatin, and resveratrol-3-α-glucoside are summarized in Table 1. All three molecules exhibit high gastrointestinal absorption. According to the ADMETLab platform, none of the compounds act as P-glycoprotein (P-gp) substrates or inhibitors. Additionally, ADMETLab predicts that RV has a probability of over 70% of achieving at least 30% oral bioavailability. In contrast, the glycosylated derivatives exhibit a high probability (>70%) of having bioavailability below 30%, indicating poor oral absorption.

**Table 1:** Summary of Resveratrol, Polydatin, and Resveratrol-3-α-glucoside Physicochemical and Predicted ADMET Properties

	**Resveratrol**	**Polydatin**	**RV-3-α-Glucoside**
**Physicochemical Properties**			
**Formula**	C_14_H_12_O_3_	C_20_H_22_O_8_	C_20_H_22_O_8_
**Molecular weight**	228.24 g/mol	390.38 g/mol	390.38 g/mol
**Number H-bond acceptors**	3	8	8
**Number H-bond donors**	3	6	6
**TPSA**	60.69 Å^2^	139.84 Å^2^	139.84 Å^2^
**Lipophilicity**			
**LogP**	2.895	1.479	0.937
**Water Solubility**			
**LogS**	–3.607	–3.313	–3.342
**Class**	Moderately soluble	Soluble	Soluble
**Pharmacokinetics**			
**Absorption**			
**GI absorption**	High	High	High
**P-gp substrate**	No	No	No
**Bioavailability (F30%)** ^a^	>70%	<70%	<70%
**Distribution**			
**PP binding**	Yes (88.6%)	Yes (83.7%)	Yes (82.7%)
**VD**	1.262 L/kg	0.848 L/kg	0.717 L/kg
**BBB permeant**	Yes	No	No
**Metabolism**			
**CYP1A2 inhibitor**	Yes	No	No
**CYP1A2 substrate**	No	No	No
**CYP2C19 inhibitor**	No	No	No
**CYP2C19 substrate**	No	No	No
**CYP2C9 inhibitor**	No	No	No
**CYP2C9 substrate**	No	No	Yes
**CYP2D6 inhibitor**	No	No	No
**CYP2D6 substrate**	Yes	No	Yes
**CYP3A4 inhibitor**	Yes	No	No
**CYP3A4 substrate**	No	No	No
**Excretion**			
**Clearance rate**	9 mL/min/kg	2.7 mL/min/kg	3.5 mL/min/kg
**Half-life (*T*_1/2_)**	∼1.5 h	∼3.1 h	∼2.8 h
**Toxicity**			
**LD_50_**	1560 mg/kg	1380 mg/kg	1380 mg/kg
**Organ toxicity**	No	Yes (Kidneys)	Yes (Kidneys)
**Toxicity end points**	No	Yes (Immunotoxicity; BBB-toxicity)	Yes (Immunotoxicity; BBB-toxicity)
**NR signaling pathways toxicity**	Yes (androgen and estrogen receptors)	No	No
**Stress response pathways toxicity**	Yes (MMP and ATAD5)	No	No
**Molecular events toxicity**	No	No	No
**Druglikeness**			
**Lipinski**	Accepted; 0 violation	Accepted; 1 violation: NHorOH > 5	Accepted; 1 violation: NHorOH > 5

a Probability of showing at least 30% bioavailability (F30%). BBB: blood–brain barrier; MMP: mitochondrial membrane potential; NR: nuclear receptor; ATAD5: ATPase family AAA domain containing protein 5; NHorOH: number of H-bond donors; RV-3-α-glucoside: resveratrol-3-α-glucoside.

All three compounds show a high probability of plasma proteins binding. The predicted volume of distribution (VD) for RV is 1.262 L/kg, while polydatin and resveratrol-3-α-glucoside display lower values of 0.848 and 0.717 L/kg, respectively. ADMETLab also suggests that RV can cross the blood-brain barrier (BBB). Meanwhile, glycosylated derivatives do not possess the capability to penetrate the central nervous system.

RV exhibits a high predicted probability of interacting with specific cytochrome P450 (CYP450) enzymes, particularly as an inhibitor of CYP1A2 and CYP3A4, and CYP2C8 isoforms and as a substrate of CYP2D6. Resveratrol-3-α-glucoside was predicted to act exclusively as substrates for CYP2C9 and CYP2D6 isoforms. Polydatin showed no interaction with the CYP450 enzymes.

Excretion predictions indicate that RV is cleared more rapidly from the system than polydatin and resveratrol-3-α-glucoside, showing a clearance rate of 9 mL/min/kg, approximately more than the double of glycosylated derivative clearance rate. Both clearance rate and volume of distribution are critical factors influencing the drug half-life and dosage frequency. Consequently, all three compounds exhibit less than 3 h of half-life, considered low. Notably, RV seems to have the shortest predicted half-life, around 1.5 h, reflecting its rapid clearance from the bloodstream.

RV seems to be less toxic compared to its glycosylated forms, exhibiting a predicted LD_50_ of 1560 mg/kg against 1380 mg/kg of both polydatin and resveratrol-3-α-glucoside (prediction accuracy of 68.07%). These values indicate that all three compounds have been predicted with toxicity classification 4 according to the Globally Harmonized System of Classification of Labeling of Chemicals (GHS), with potential to cause acute oral toxicity. Although RV showed to be safer than its glycosylated forms, the aglycone demonstrates high probability of causing toxicity to androgen and estrogen nuclear receptor signaling pathways, as well as to cause disturbance of mitochondrial membrane potential and DNA replication. Glycosylated derivatives exhibited a probability of 73% to be nephrotoxic, in addition to showing potential to cause immunotoxicity and chemical induced BBB toxicity.

### 3.2 Target Identification and Functional Enrichment Analysis of RV and Its Glycosylated Derivatives in PD

From the CTD database, 7,250 targets were retrieved for RV and 69 for polydatin. SwissTargetPrediction identified 100 predicted targets for each of the three compounds (RV, polydatin, and resveratrol-3-*O*-α-glucoside). No targets for resveratrol-3-*O*-α-glucoside were listed in the CTD database. After merging and removing duplicates, the final data set consisted of 7,275 RV targets, 163 polydatin targets, and 100 resveratrol-3-*O*-α-glucoside targets. Across these sets, 67 targets were found to be common to all three molecules (Figure 2A). Additionally, a total of 4870 PD-related targets were collected from the GeneCards database. Both final target sets were intersected, revealing 51 potential common targets of RV and its glycosylated derivatives in the context of PD (Figure 2B), including catalytic proteins, signaling molecules, and transporters (Figure 2C).

**Figure 2:**
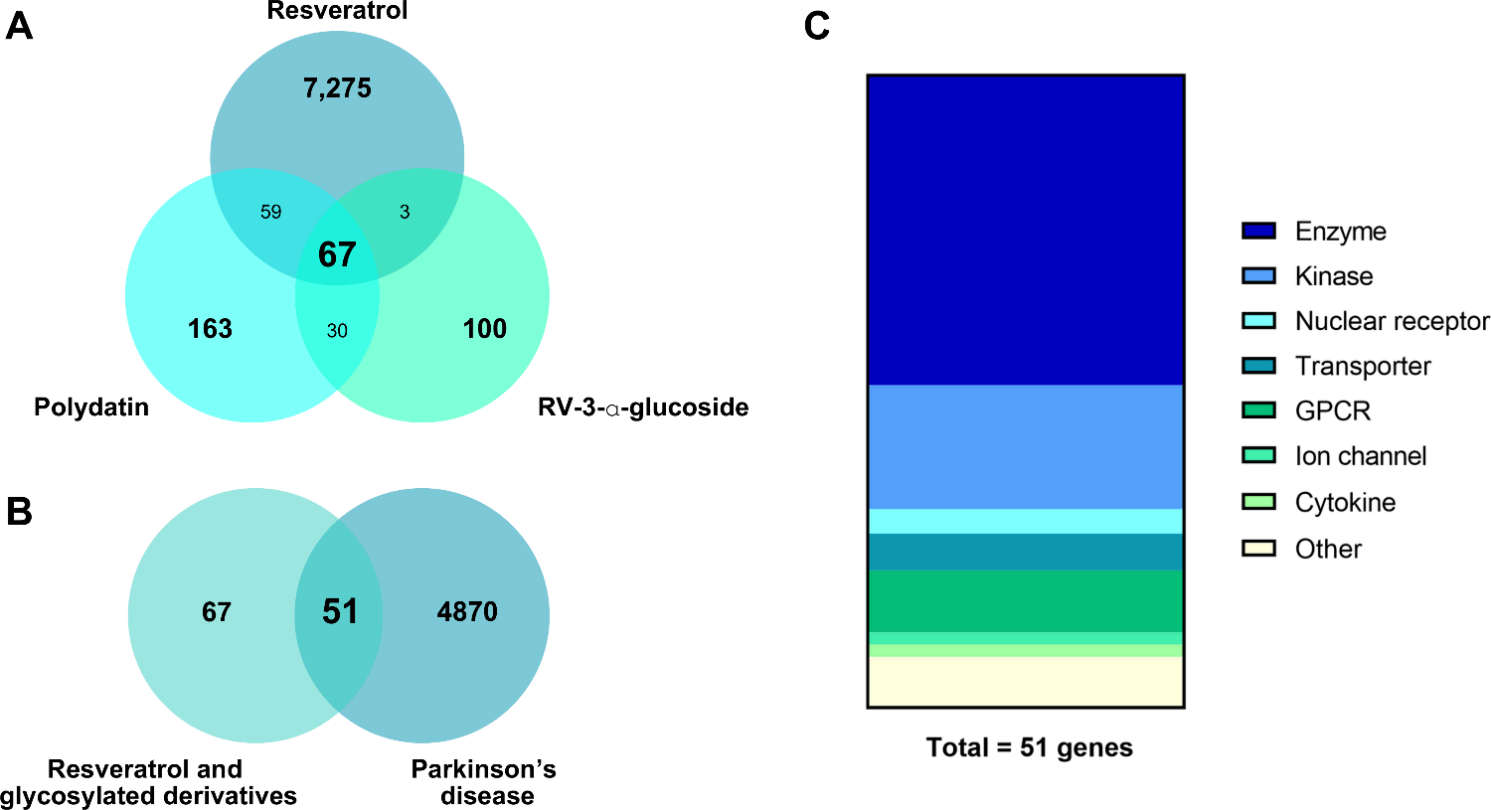
Target identification and intersection analysis of resveratrol, polydatin, and resveratrol-3-α-glucoside in the context of PD. (A) Venn diagram showing the number of predicted targets for each compound, obtained from CTD and SwissTargetPrediction databases. (B) Intersection between compound targets and PD-related targets retrieved from the GeneCards database, identifying 51 common targets. (C) Classification of the identified common targets based on their molecular functions, including catalytic proteins, signaling molecules, and transporters.

GO enrichment analysis identified a total of 186 biological functions and cellular roles significantly associated (*p* ≤ 0.05) with the 51 genes set of RV, polydatin, and resveratrol-3-α-glucoside in PD. Among these, 127 biological processes, 27 cellular components, and 32 molecular functions were annotated. Biological processes included those related to apoptosis and cell survival, immunity and inflammation, endocrine functions, cell signaling, development and cell differentiation, metabolic and catabolic processes, cardiovascular and vascular functions, and transcriptional and post-transcriptional regulation, as well as responses to environmental and toxic stimuli. Cellular components involved in the set of genes of targets included membrane structures, intracellular organelles, extracellular structures, protein complexes, and vesicles. Molecular functions prevalent in the data set included enzymatic activities of kinases and phosphorylation, peptidases and proteolysis, and oxidoreductases, as well as their regulation and interactions with structural proteins. Functions such as glucose transport, metal ion and nucleotide binding, and cellular interaction and signaling were also identified. To provide deeper insight into the GO enrichment of these 51 target genes, the 15 most significant entries for each component, based on p-values, are present in Figure 3A.

**Figure 3:**
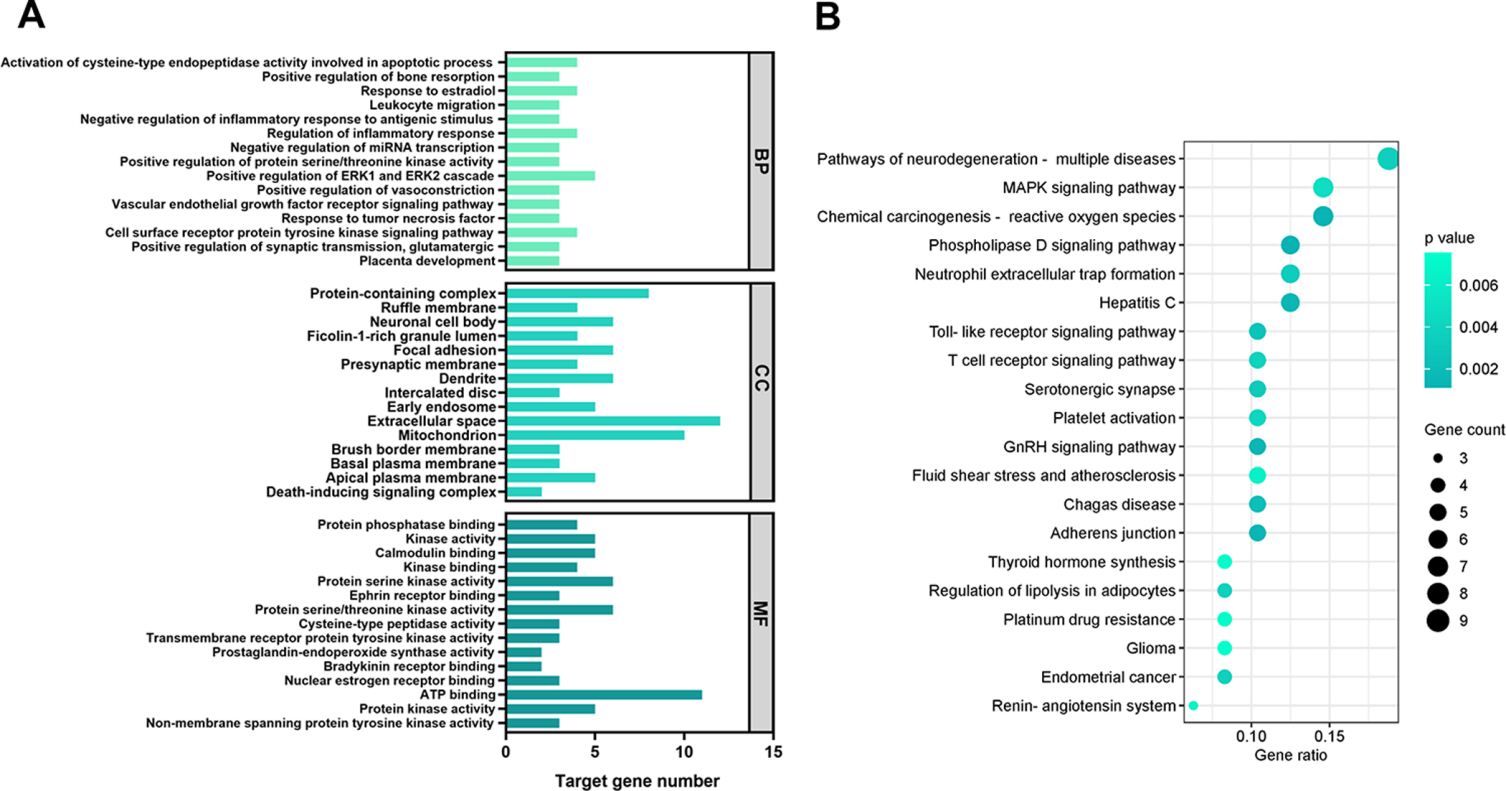
GO and KEGG pathway enrichment analyses of resveratrol, polydatin, and resveratrol-3-α-glucoside targets in PD. (A) GO enrichment analysis highlighting the 15 most significant biological processes, cellular components, and molecular functions associated with the 51 common target genes. (B) KEGG pathway enrichment analysis displaying the top 20 most significantly enriched pathways, providing insights into the molecular mechanisms through which resveratrol, polydatin, and resveratrol-3-α-glucoside may exert their effects in PD. BP: biological processes; CC: cellular compartment; MF: molecular function.

KEGG pathway enrichment analysis revealed a list of 113 signaling pathways. Of these, 53 pathways were significantly enriched from the initial set of 51 target genes (*p* ≤ 0.05). The top 20 pathways with the most significant p-values were plotted in a bubble chart (Figure 3B).

### 3.3 PPI Network and Key Target Identification

When the list of common targets was uploaded into the STRING platform, a PPI network was generated consisting of 50 nodes and 326 edges, representing the proteins and their predicted functional interactions, respectively. The blue intensity of the node reflects the degree value, with nodes at the center indicating higher degree values. The edge thickness corresponds to the combined score, indicating the predicted confidence level of the interactions (Figure 4). No direct connections were identified for the SLC28A2 and DYRK1A genes, which were positioned at the left extremity of the network. Topological analysis of the PPI network revealed an average node degree of 13. By combining the average degree (≥13), betweenness centrality (≥0.03), and closeness centrality values (≥0.4), the 50 targets were screened for key interactions. Eleven targets were identified as potential key targets that may play a role in functions related to the molecular mechanisms of RV and its glycosylated derivatives in PD (Table 2).

**Figure 4:**
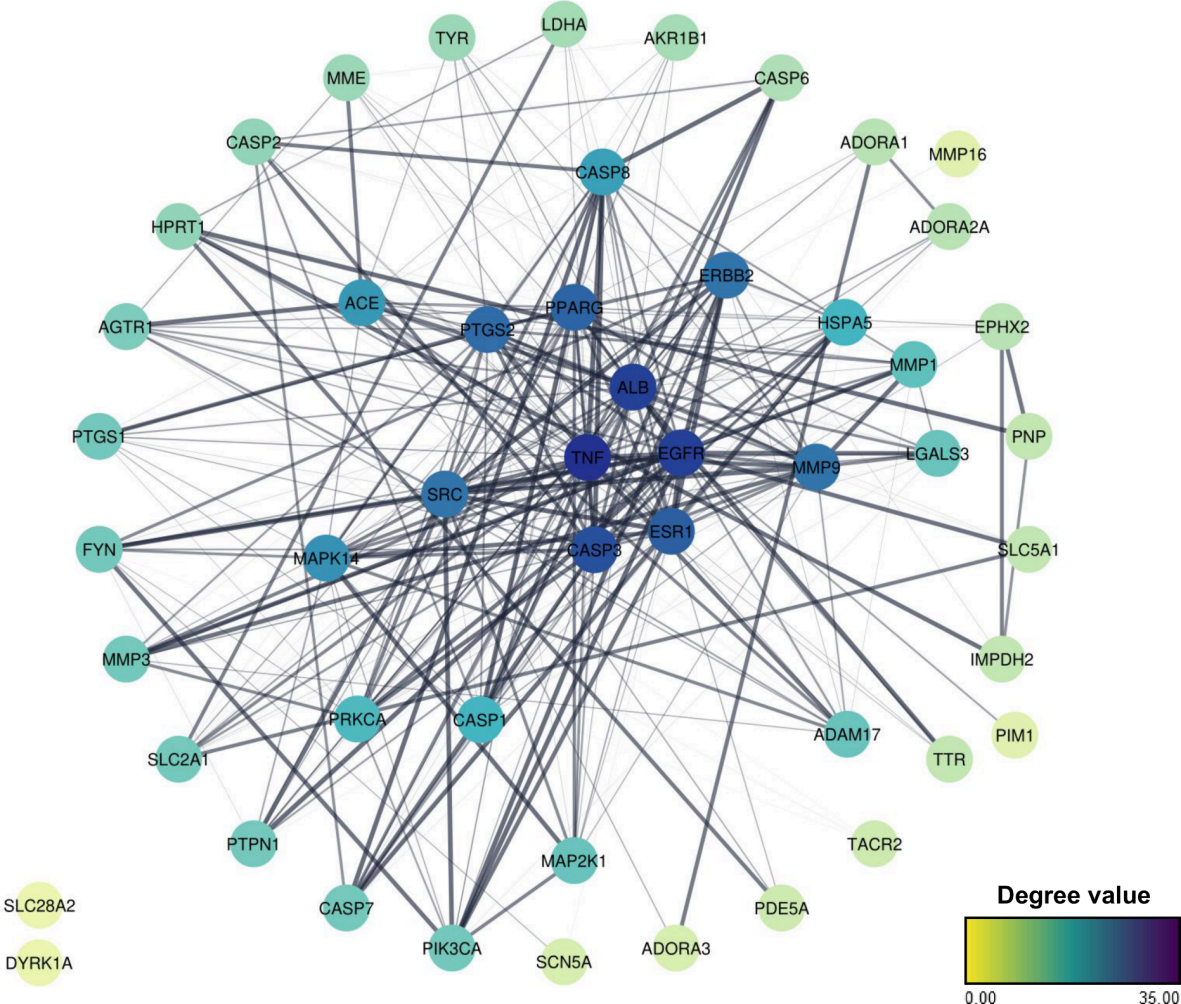
PPI network of resveratrol, polydatin, and resveratrol-3-α-glucoside targets in PD. The network includes 50 proteins and 326 functional interactions, with node color intensity indicating connectivity and edge thickness representing interaction confidence. SLC28A2 and DYRK1A showed no direct connections.

**Table 2:** Protein–Protein Interaction Network Results with the Potential Key Targets of Resveratrol, Polydatin, and Resveratrol-3-α-glucoside in Parkinson’s Disease^a^

Gene	Degree value	Betweenness centrality	Closeness centrality
TNF	35	0.092	0.797
EGFR	33	0.110	0.770
ALB	33	0.069	0.770
CASP3	31	0.054	0.746
ESR1	29	0.096	0.723
PPARG	28	0.051	0.712
PTGS2	27	0.061	0.701
ERBB2	26	0.062	0.681
SRC	26	0.063	0.681
ACE	21	0.033	0.627
MAP2K1	13	3.802	0.573

a Screening parameters: degree value (≥13); betweenness centrality (≥0.03); closeness centrality (≥0.4).

### 3.4 Molecular Docking Analysis of RV and Glycosylated Derivatives with PD-Related Targets

To investigate if glycosylation influences the affinity and binding characteristics of RV, polydatin, and resveratrol-3-α-glucoside with target proteins involved in PD, molecular docking simulations were performed. Through network pharmacology analysis, we identified 11 core proteins that might be key targets of RV, polydatin, and resveratrol-3-α-glucoside in PD. After molecular docking verifications, glycosylation appears to enhance binding affinity compared to the aglycone form of RV, showing higher binding scores in ten of the 11 receptors analyzed. Furthermore, TNF-α, PPARγ, and ERBB2 showed the best degree of binding with RV and glycosylated derivatives. Binding affinity between RV and its glycosylated derivatives and the 11 core target proteins identified through PPI network analysis are summarized in Figure 5 and Table S2. The lower the docking score (more negative), the stronger the affinity between the ligand molecule and the target protein. For comparative purposes, Figure 5 also includes the binding energies of the ligands cocrystallized with each target protein in their respective PDB structures. These “original” ligands were not part of the set of tested RV and glycosylated derivatives but rather corresponded to the molecules experimentally determined as bound to the receptor in the crystallographic complexes. The inclusion of these data provides methodological control and constitutes a validation step of the docking approach, enabling the evaluation of the accuracy and consistency of the docking protocol through a direct comparison between the predicted affinities of the reference ligands and those of the experimentally studied compounds. RV and its glycosylated derivatives showed good affinity for tumor necrosis factor-α (TNF-α), peroxisome proliferator-activated receptor γ (PPARγ), and human epidermal growth factor receptor 2 (ERBB2) receptors with docking scores ranging from −9.1 to −9.6 kcal/mol. Detailed diagrams of ligand–receptor interactions for these targets are provided. Further details of the other targets are available in the Supporting Information (Figures S1–8). Hydrogen bonds and hydrophobic interactions were the main interaction types. Glycosylated forms showed higher values of docking score compared to the aglycone form of RV. Polydatin demonstrated the best binding energy for most targets, suggesting potentially superior performance compared to that of RV and resveratrol-3-α-glucoside. For each receptor system, the lowest-energy docking pose was selected to represent the ligand–protein interaction model shown in Figures 6–11. These conformations correspond to the best-scoring results from the docking analysis (Figure 5) and were chosen to depict the main binding mode and the relevant intermolecular interactions within the active sites.

**Figure 5:**
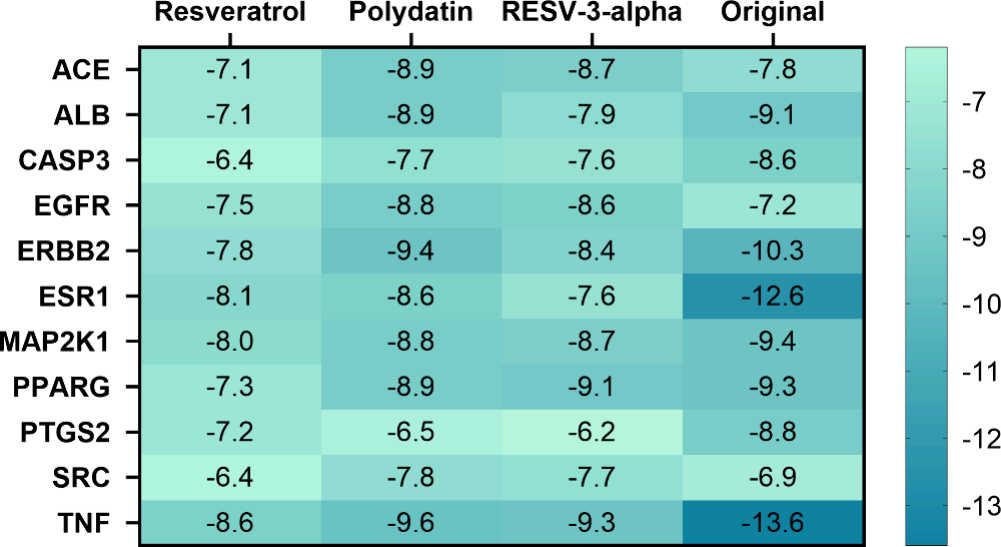
Binding energies of resveratrol, polydatin, and resveratrol-3-α-glucoside with the 11 core target proteins identified in the PPI network analysis. The column labeled “original” represents the cocrystallized ligand present in the experimental structure deposited in the PDB database; these reference ligands in the analysis serve as a methodological control and validation step to verify the reliability of the docking procedure. The lower the binding energy values, the stronger the molecular interactions.

**Figure 6:**
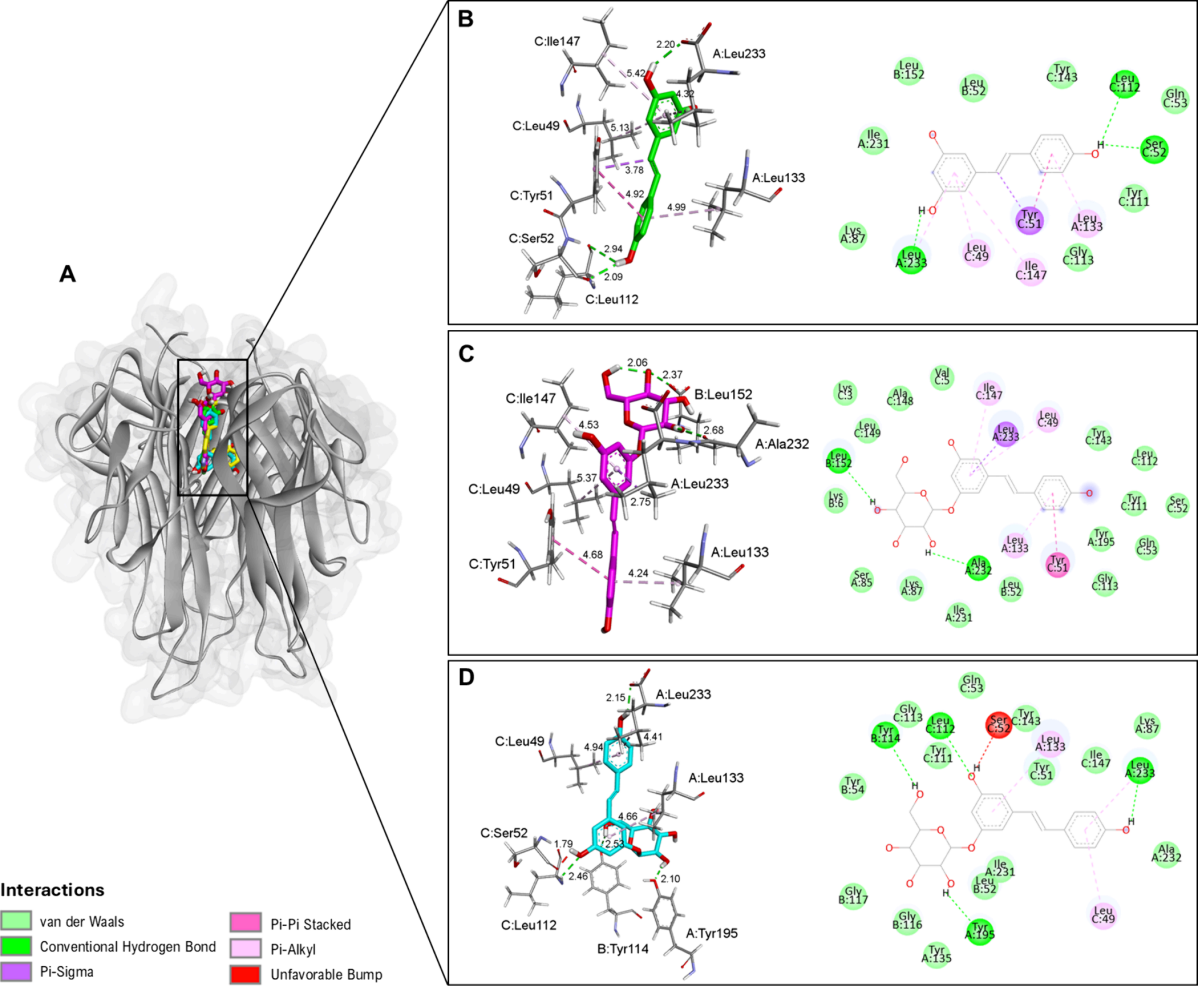
(A) Binding interactions of resveratrol, polydatin, and resveratrol-3-α-glucoside within the TNF-α receptor active site. Resveratrol (B), polydatin (C), and resveratrol-3-α-glucoside (D) molecular interactions, with the panels on the left corresponding to the 3D structures and those on the right corresponding to the 2D diagrams. Letters before residue names indicate the corresponding protein chain in the PDB structure.

Figure 6 shows the binding interactions between RV, polydatin, and resveratrol-3-α-glucoside in the active site of TNF-α receptor homotrimer structure (chain A, B, C), with docking scores of −8.6, −9.6, and −9.3 kcal/mol, respectively. RV and resveratrol-3-α-glucoside both form conventional hydrogen bonds with A:Leu233 and C:Leu112 residues. In general, RV forms three hydrogen bonds (A:Leu233, C:Leu112, C:Ser52) (Figure 6B), while polydatin presents two (A:Ala232, B:Leu52) (Figure 6C) and resveratrol-3-α-glucoside forms four (A:Leu233, A:Tyr195, B:Tyr114, C:Leu112) (Figure 6D). Resveratrol-3-α-glucoside also showed a sterically unfavorable interaction with the C:Ser52 residue, which may impact its binding affinity to the TNF-α receptor (Figure 6D). Additionally, all three molecules exhibit hydrophobic interactions in various amino acid residues in the TNF-α active site, including Pi-Alkyl, Pi-Sigma, and Pi-Pi Stacked interactions that often enhance the stability of ligand–receptor complexes (Figure 7). Although polydatin exhibited fewer conventional hydrogen bonds with TNF-α compared to RV and resveratrol-3-α-glucoside, it showed extensive hydrophobic contacts involving residues such as A:Leu49, A:Leu133, A:Leu233, and C:Tyr111 and aromatic stacking with C:Tyr51 (Figure 7). These interactions likely compensate for the smaller number of hydrogen bonds, stabilizing the ligand–protein complex and resulting in a more favorable binding energy.

**Figure 7:**
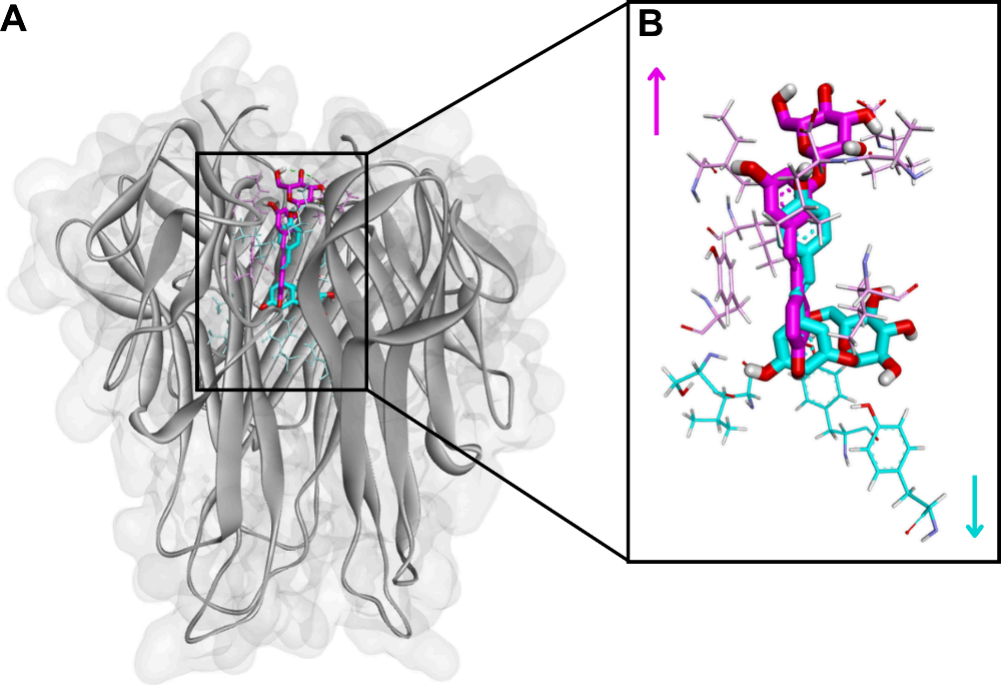
(A) Comparison of α- and β-configurations of the glycosidic bond. (B) The α-form of resveratrol-3-α-glucoside (thick structure in cyan blue) positions the sugar moiety inward to the protein pocket, modifying the arrangement of hydrophobic contacts (thin structure in cyan blue), whereas the β-form of polydatin points the sugar outward (thick structure in pink), yielding distinct hydrogen-bonding orientations (thin structure in pink).

The comparative analysis of the glycosylated derivatives within the TNF-α binding site revealed a clear difference in the spatial orientation of the glucose moiety, depending on the type of glycosidic bond. In resveratrol-3-α-glucoside, the α-glycosidic linkage positions the glucose unit in the same plane and direction as the aromatic rings of the RV scaffold, resulting in a more compact and inward-oriented conformation within the binding site. In contrast, in polydatin, the β-glycosidic linkage projects the glucose unit outward away from the aromatic core, exposing it to the solvent interface. This opposite orientation markedly influences how both molecules occupy the binding pocket, affecting the accessibility of hydrogen-bond donors and acceptors and the overall distribution of hydrophobic and polar contacts. Consequently, the α- and β-configurations determine distinct spatial accommodation patterns, which can modulate the stability and specificity of ligand–receptor interactions, as illustrated in Figure 7.

Interactions with A:Leu233 and C:Leu112 of TNF-α may be important for complex stabilization, as leucine, being an apolar amino acid, promotes hydrophobic interactions and hydrogen bonding, especially with compounds that contain aromatic rings, such as RV and its glycosylated derivatives. Although resveratrol-3-α-glucoside formed favorable interactions with A:Leu233 and C:Leu112, its binding affinity could be slightly decreased due to the conformational adjustments required to accommodate these interactions as well as the presence of an unfavorable bump with C:Ser52 residue, located near C:Leu112, which could impact complex stability and cause a minor decrease in binding affinity of resveratrol-3-α-glucoside compared to polydatin. Polydatin, on the other hand, established more stable hydrophobic interactions, demonstrating a slight energy advantage (Figure 8).

**Figure 8:**
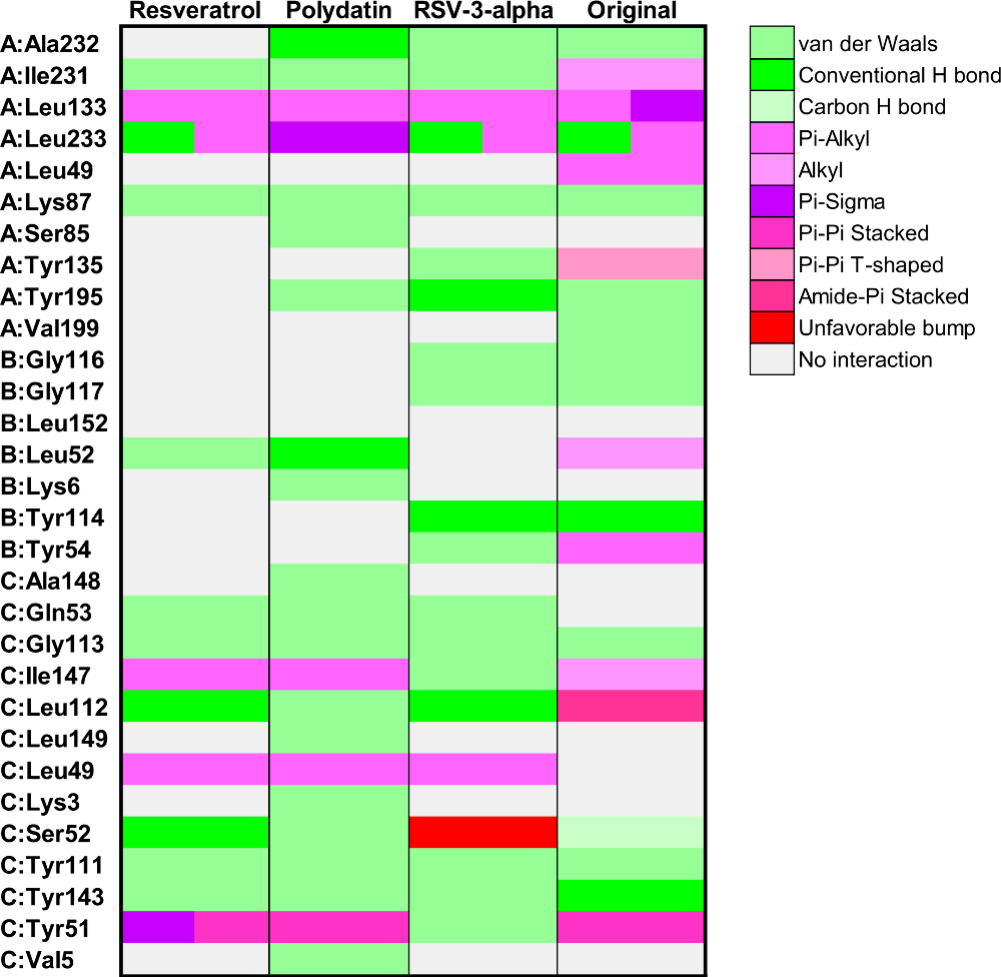
Heatmap illustrating the interactions between TNF-α protein residues and resveratrol, polydatin, and resveratrol-3-α-glucoside. The color gradient represents different types of interactions, as detailed in the right panel. Letters before residue names indicate the corresponding protein chain in the PDB structure.

Binding interactions of all three compounds and PPARγ target (Figure 9A) revealed the formation of hydrogen bonds into the monomer protein structure; notably, polydatin showed three hydrogen interactions (Figure 9C). Alternatively, RV and resveratrol-3-α-glucoside both established Pi-Cation interactions with the Arg288 residue (Figure 9B,D). Similar hydrophobic contacts near the RV scaffold, including Ile341, Cys285, and Leu330 amino acid residues, were observed for all three compounds (Figure 10). Among the tested ligands, resveratrol-3-α-glucoside presented the most favorable docking score (−9.1 kcal/mol). Its interaction profile included multiple van der Waals contacts with Leu353, Met364, and Leu333, as well as Pi-Alkyl and Pi-Cation interactions involving Arg288 and Val339, which reinforce the anchoring of its aromatic core (Figure 10). Such a balance between polar and apolar interactions likely accounts for the lower energy score observed for resveratrol-3-α-glucoside.

**Figure 9:**
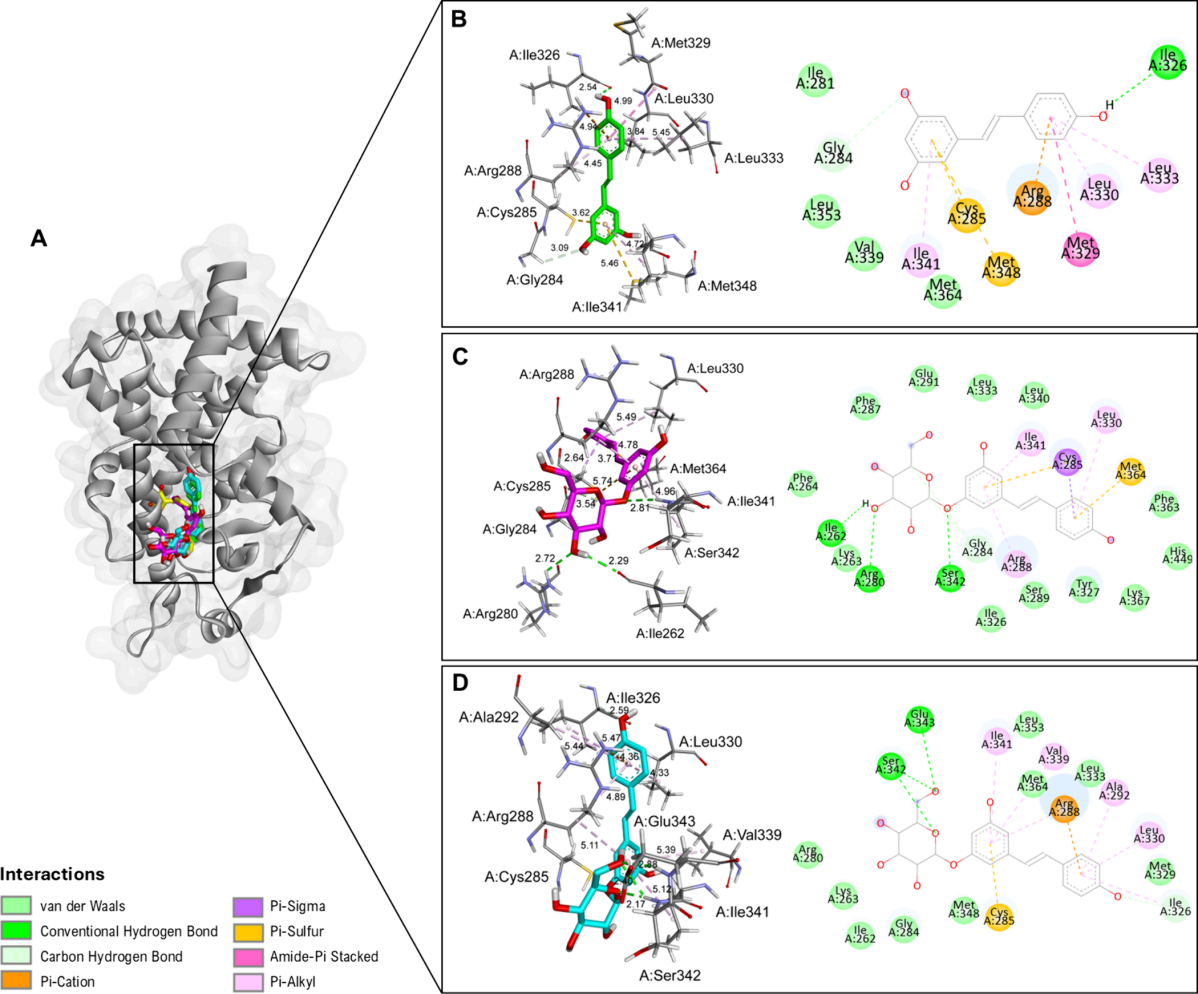
(A) Binding interactions of resveratrol, polydatin, and resveratrol-3-α-glucoside within the PPARγ receptor active site. Resveratrol (B), polydatin (C), and resveratrol-3-α-glucoside (D) molecular interactions, with the panels on the left corresponding to the 3D structures and those on the right corresponding to the 2D diagrams. Letters before residue names indicate the corresponding protein chain in the PDB structure.

**Figure 10:**
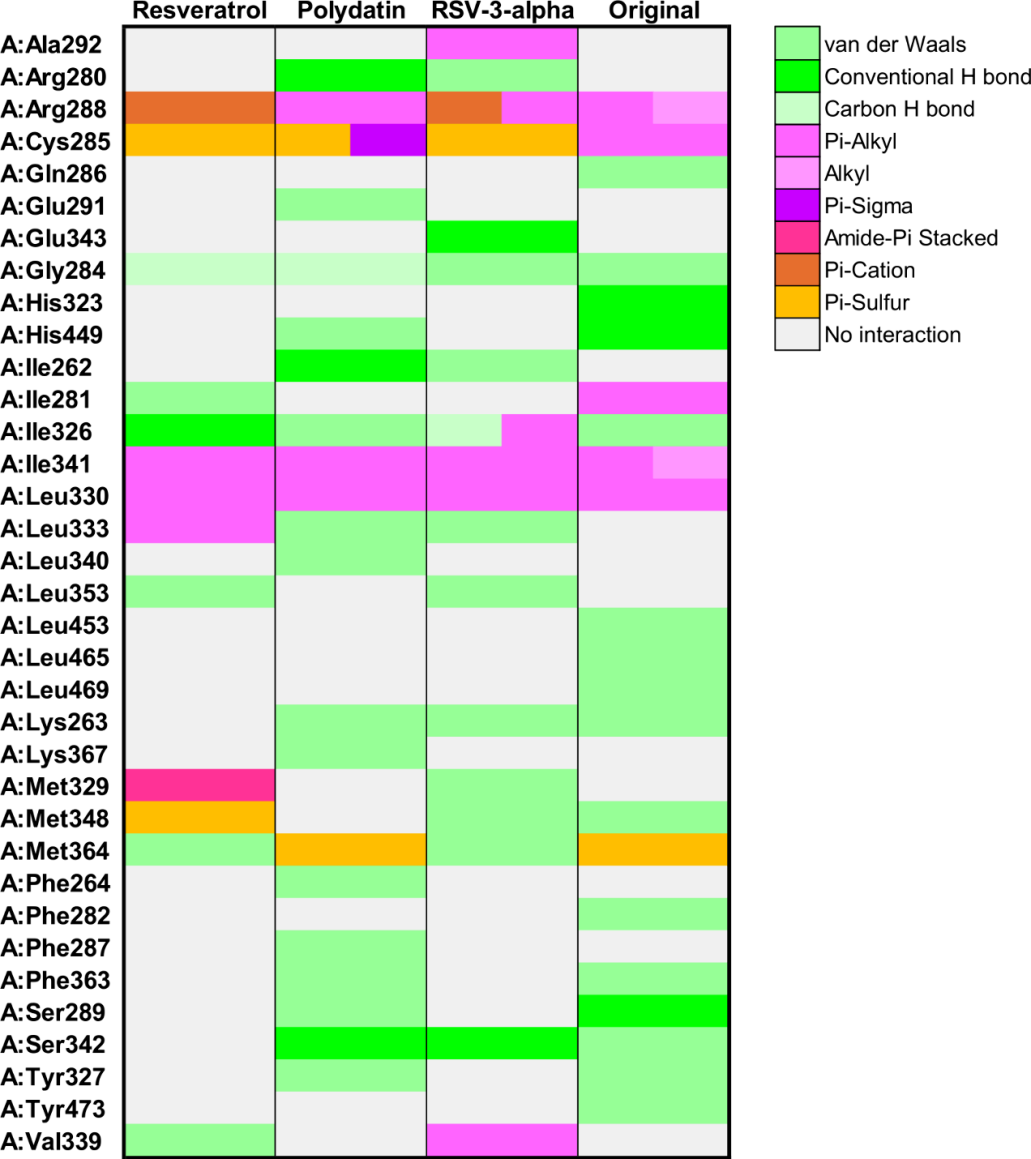
Heatmap illustrating the interactions between PPARγ protein residues and resveratrol, polydatin, and resveratrol-3-α-glucoside. The color gradient represents different types of interactions, as detailed in the right panel. Letters before residue names indicate the corresponding protein chain in the PDB structure.

The glycosylated derivatives displayed additional stabilization due to the extra hydroxyl groups in the glucose moiety, which facilitated hydrogen-bond formation and overall complex stability. All three ligands were located near the β-sheet region of PPARγ, a well-described binding site for partial agonists of PPARγ receptor.^31,32^ Notably, only the glycosylated derivatives formed hydrogen bonds with the critical Ser342 residue, while multiple hydrophobic contacts involving the aromatic rings of the RV scaffold were detected for all ligands. Interactions with Cys285, a key residue in the PPARγ active site,^33^ were particularly significant: RV and resveratrol-3-α-glucoside formed Pi-Sulfur interactions with ring A, whereas polydatin exhibited a Pi-Sulfur contact in ring A and an additional Pi-Sigma interaction in ring B. These noncovalent contacts contribute to a more flexible and dynamic ligand attachment, enabling reversible binding. Moreover, the Pi-Cation interaction observed between Arg288 and both RV and resveratrol-3-α-glucoside further enhances affinity by combining electrostatic attraction and stabilization forces.^34^

Considerable differences were observed in the interactions with the ERBB2 receptor monomer protein structure (Figure 11). Consistent with the overall trend of docking results, glycosylated derivatives exhibited binding affinities stronger than those of the aglycone RV, with polydatin showing the most favorable docking score (−9.4 kcal mol^–1^), followed by resveratrol-3-α-glucoside (−8.4 kcal mol^–1^) and RV (−7.8 kcal mol^–1^).

**Figure 11:**
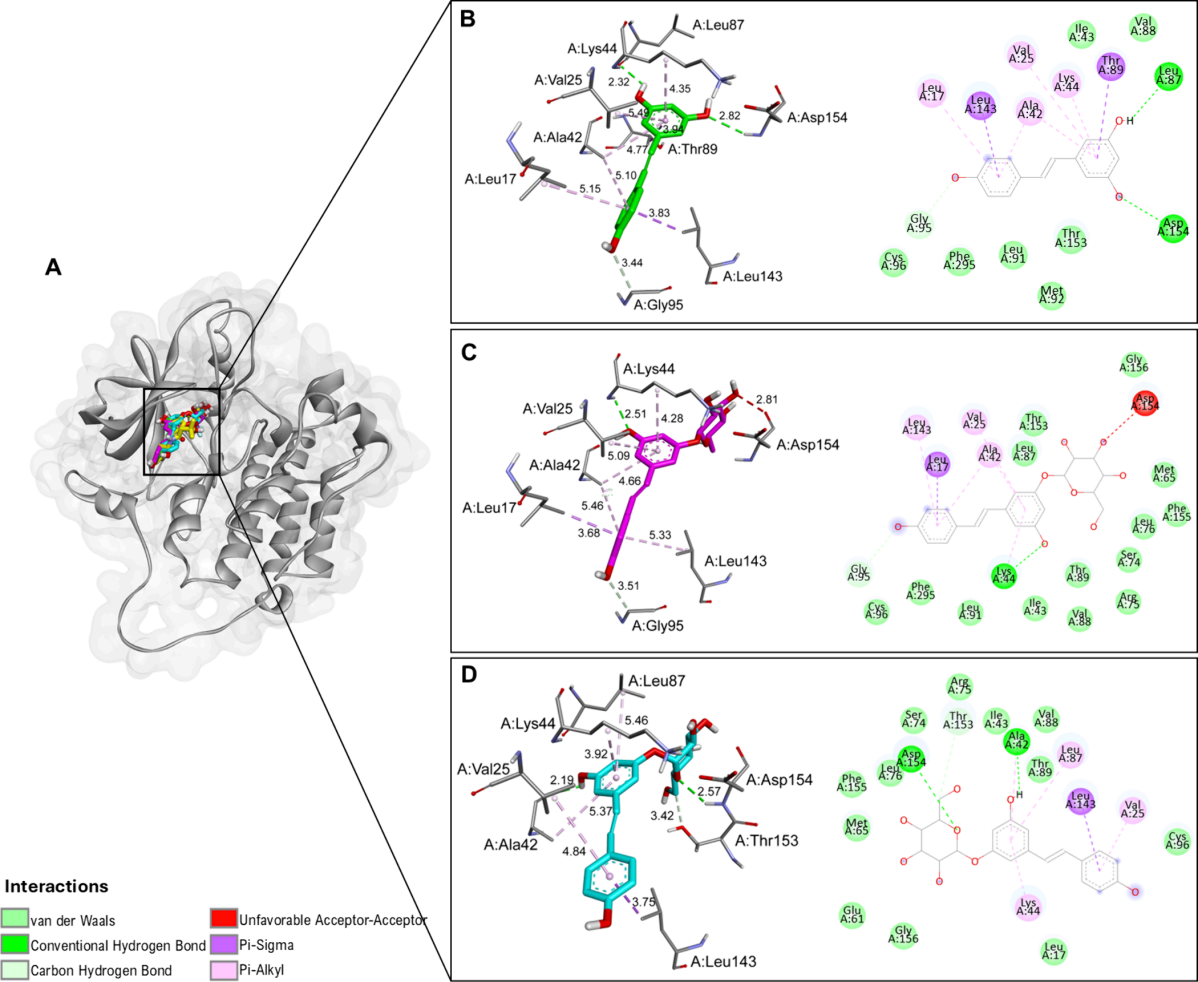
(A) Binding interactions of resveratrol, polydatin, and resveratrol-3-α-glucoside within the ERBB2 receptor active site. Resveratrol (B), polydatin (C), and resveratrol-3-α-glucoside (D) molecular interactions, with the panels on the left corresponding to the 3D structures and those on the right corresponding to the 2D diagrams. Letters before residue names indicate the corresponding protein chain in the PDB structure.

Both RV and resveratrol-3-α-glucoside formed hydrogen bonds with Asp154, whereas polydatin displayed an unfavorable acceptor–acceptor contact with the same residue, likely producing mild repulsion and a less stable local geometry (Figure 11C). Despite this unfavorable interaction, polydatin established an extensive network of hydrophobic and van der Waals contacts involving residues Leu143, Val25, Ala42, and Lys44, as well as Pi-Sigma and Pi-Alkyl interactions typical of apolar stabilization within the binding pocket (Figure 12). The accumulation of these dispersion and hydrophobic forces effectively compensated for the reduced number of hydrogen bonds, explaining its lowest binding energy and highest overall complex stability among the three ligands.

**Figure 12:**
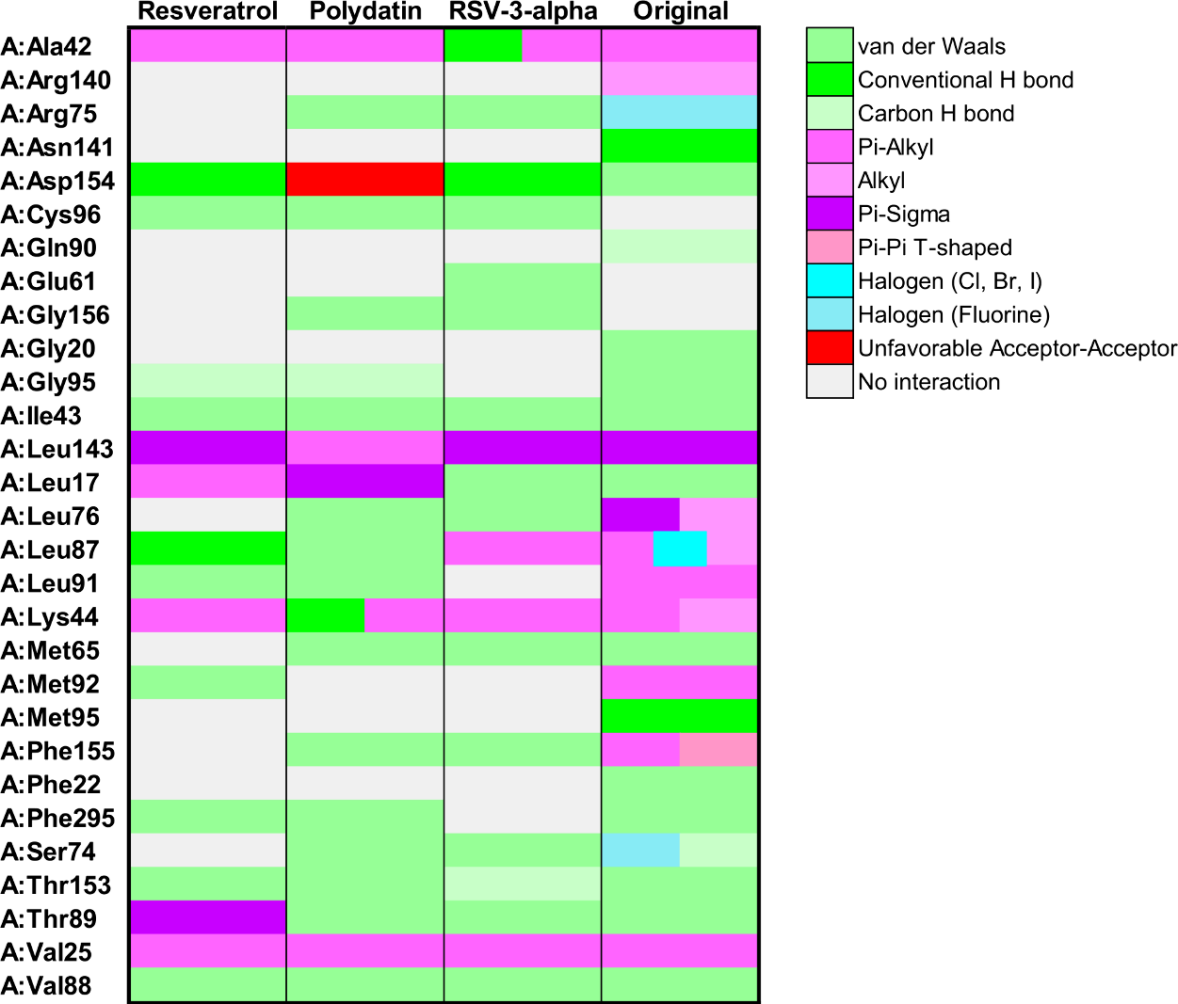
Heatmap illustrating the interactions between ERBB2 protein residues and resveratrol, polydatin, and resveratrol-3-α-glucoside. The color gradient represents different types of interactions, as detailed in the right panel. Letters before residue names indicate the corresponding protein chain in the PDB structure.

## 4 Discussion

Understanding the molecular mechanisms involved in the interaction of bioactive compounds with therapeutic targets is essential for the development of new therapeutic strategies. In this study, we employed *in silico* approaches to investigate the impact of RV glycosylation on its pharmacokinetic properties and molecular interactions with proteins associated with PD. Our findings indicate that the introduction of glycosyl residues can modulate the binding affinity and the stability of ligand–receptor complexes, influencing potential neuroprotective effects. Additionally, we explored how the type of glycosidic bond (α or β) may impact these interactions, providing insights into the structural role of glycosylation in the bioactivity of RV derivatives. In the following sections, we discuss the results obtained and their implications in the context of optimizing natural compounds for PD treatment.

Our study found that glycosylation appears to decrease the predicted bioavailability of glycosylated derivatives compared to RV aglycone. Experimental findings have already shown that polydatin exhibits 3–4 times higher bioavailability than RV.^35^ Although *in silico* methods are valuable tools for predicting the properties of compounds, they may not capture the full complexity of *in vivo* absorption and metabolism.^36^ This is particularly relevant for compounds with structural modifications such as glycosylation, which can alter bioavailability in a complex manner. Glycosylation can facilitate absorption through specific transporters, but it can also hinder it by increasing the polarity of the compound.^37^ Bioavailability can also be influenced by factors such as the volume of distribution, clearance rate, and half-life of a compound. RV’s high volume of distribution and clearance rate, combined with its shorter half-life compared to glycosylated derivatives, may reflect its hydrophobic structure, which facilitates penetration through lipidic cell membranes but makes it more vulnerable to enzymatic metabolism and rapid elimination. Conversely, the glycosidic moiety in polydatin and resveratrol-3-α-glucoside increases their polarity, suggesting that the glycosylated derivatives remain longer in plasma by reducing distribution, which combined with better absorption and lower susceptibility to enzymatic oxidation can contribute to a more stable bioavailability.^19,22,38^

The ability to cross the BBB is a determining factor for potential compound efficacy in the treatment of neurodegenerative diseases, such as PD.^39^ Our *in silico* predictions suggest that polydatin and resveratrol-3-α-glucoside exhibited significant differences in their BBB permeability. While RV, which possesses a hydrophobic structure and low molecular weight, was shown to cross the BBB, its glycosylated derivatives tend to be more hydro soluble, which could compromise their passive diffusion. Nevertheless, glucose transporters expressed in brain tissue, such as GLUT1, may mediate the passage of these glycoconjugate compounds across the BBB.^40,41^

RV showed a higher LD_50_ compared with glycosylated derivatives, being generally less toxic. Differently, *in vivo* studies have shown that polydatin has lower toxicity compared to the aglycone form.^19,42,43^ *In silico* tools generally are based on computational models and databases of chemical structures, where the toxicity prediction can mainly consider the compound chemical structure and its physicochemical properties, without taking into account the interactions with transporter proteins, metabolization enzymes, and pharmacokinetic factors such as distribution and excretion, as mentioned above.^44,45^ These facts highlight the importance of checking the literature, when available, in parallel to prediction of *in silico* platforms.

The enzymes of the CYP450 system are responsible for the biotransformation of multiple drugs. Investigating possible interactions with drug-metabolizing enzymes is crucial during the early stages of drug discovery and development. These isoenzymes can be inhibited or induced by compounds, the main reason for unanticipated adverse effects occurring due to drug–drug interactions.^46,47^ Our results show that RV can inhibit CYP450 enzymes, which is in line with the literature already published.^48–50^ Inhibiting CYP450 enzymes can decrease the metabolization of other drugs that depend on the same pathway to be metabolized, increasing this drug concentration and leading to important adverse drug interactions.^47^ RV and glycosylated derivatives can also act as substrates of CYP2D6 and CYP2C9, respectively. Being metabolized by these CYP450 enzymes can result in individual variations of biological responses when compounds are administered.^51^ When coadministered with an inhibitor (e.g., fluoxetine or valproic acid), RV and glycosylated derivatives can be slowly metabolized, increasing their half-life and bioavailability, which may consequently potentiate their therapeutic effects. However, if RV and its glycosylated forms are coadministered with CYP2D6 and CYP2C9 inducers (e.g., dexamethasone or corticosterone, and phenobarbital or carbamazepine, respectively) they would be metabolized even faster, reducing their therapeutic efficacy.^52,53^

To be considered a promising drug candidate, the compound should present some essential pharmacokinetic characteristics such as high absorption rate, adequate distribution among tissues and organs reaching the therapeutic target, controlled metabolization, medium half-life, efficient excretion and good bioavailability values.^54^ Taking into account this aimed pharmacokinetic profile, RV exhibited a promising predicted pharmacokinetic profile compared with its glycosylated derivatives. As mentioned before, RV also shows the ability to cross the BBB, a crucial factor for addressing therapeutic effects on PD. Moreover, RV’s relatively low predicted toxicity profile, when compared to its glycosylated derivatives, further enhances its potential as a promising candidate for development into a therapeutic agent for PD.

For a deeper understanding of the molecular mechanisms of RV and its glycosylated derivatives, it is important to investigate their interactions with specific biological targets, especially in the context of pathways related to neuroprotection and neurodegeneration on PD. RV and glycosylated derivative neuroprotective activities seem to be related to the modulation of apoptotic processes as well as cell proliferation and survival, as indicated in the GO enrichment analysis. The RV scaffold can act as a phytoestrogen, promoting responses linked to estrogen receptors that can potentialize protective effects of ERK signaling pathway.^55–57^ These processes appear to occur in extracellular space, mitochondrion, and protein-containing complexes, possibly related to molecular functions such as the phosphorylation of kinase cascades. KEGG analysis suggests modulation of pathways involved in neurodegeneration across multiple diseases, damage induced by ROS, and the phospholipase D signaling pathway, which may play a key role in RV, polydatin, and resveratrol-3-α-glucoside therapeutic actions. These findings align with PD physiopathology pathways already well described in the literature such as the involvement of oxidative stress, neuroinflammation, and mitochondrial dysfunction.^58^

TNF-α is a pro-inflammatory cytokine implicated in the pathogenesis and progression of PD. Elevated levels of TNF-α have been found in the serum, cerebrospinal fluid, and brain tissue of PD patients.^59,60^ As a key inflammatory mediator, targeting neuroinflammation through the modulation of TNF-α has emerged as a potential therapeutic alternative for mitigating neurodegeneration.^61^ Inhibition of TNF-α by RV and polydatin is already described in the literature.^62–67^ Different experimental studies have investigated anti-inflammatory activity and neuroprotective effects of RV and polydatin in the context of PD through decreasing TNF-α levels and expression.^68–72^

Our molecular docking analysis results revealed that RV, polydatin, and resveratrol-3-α-glucoside exhibit good affinity for TNF-α. Among these compounds, glycosylated derivatives showed the lowest binding energy, suggesting greater complex stability. This increase in stability is possibly attributed to the presence of additional hydroxyl groups in the glucose moiety, which favored additional interactions through the formation of hydrogen bonds and hydrophobic contacts.^73,74^ However, subtle differences in molecular conformation and accessibility to the active site suggest that glycosylation could have an influence on the complex stability. The distinct spatial orientations of the α- and β-glycosidic linkages appear to play important roles in stabilizing the ligand–TNF-α complexes. Although both glycosylated derivatives exhibited stronger binding affinities than the aglycone RV, their structural differences suggest complementary modes of binding rather than a simple energy advantage. This may explain why glycosylated derivatives can maintain a high affinity despite conformational constraints imposed by the sugar moiety. The observed influence of stereochemistry on molecular orientation underscores how minor structural changes, such as the inversion between α- and β-glycosidic bonds, can modulate receptor accessibility and recognition surfaces.^75^ Such variations could impact the dynamic behavior of the complexes, potentially translating into differences in downstream biological responses and anti-inflammatory activity. Overall, these findings suggest that glycosylation modulates the mode of binding of RV derivatives to TNF-α, optimizing complex stabilization through a balance of polarity, flexibility, and shape complementarity.

PPARγ is a ligand-activated transcription factor that can influence the expression and activity of a variety of targets in different signaling networks. It regulates mitochondrial function, immune responses, redox balance, and the metabolism of sugar and lipids.^76,77^ RV is a well-known activator of SIRT1, a major pathway with bidirectional activity that can indirectly modulate PPARγ. SIRT1 acts deacetylating PGC-1α, a coactivator of PPARγ, thereby stimulating the transcription of genes related to mitochondrial biogenesis, energy metabolism, and antioxidant defense, including Nuclear factor erythroid 2‑related factor 2 (Nrf2).^78,79^ Additionally, PPARγ activation can inhibit the Nuclear Factor kappa‑light‑chain‑enhancer of activated B cells (NF-κB), a key regulator of pro-inflammatory gene expression. Its inhibition reduces the production of inflammatory mediators such as TNF-α, interleukin 6 (IL-6), and cyclooxygenase-2 (COX-2), and decreases microglia activation, containing neuroinflammation.^80^ Consequently, targeting PPARγ could be a promising therapeutic strategy for PD, as its activation may modulate important pathways involved in mitochondrial function, antioxidant defense, and neuroinflammation, potentially protecting dopaminergic neurons from degeneration.

The strong binding affinities observed for resveratrol-3-α-glucoside and polydatin toward the PPARγ receptor suggest that glycosylation enhances accommodation and stability within the binding pocket. The orientation of the glucose moiety appears to facilitate an optimal hydrogen-bonding network and improve the distribution of polar and apolar contacts, contributing to a more stable ligand–receptor complex. In particular, interactions involving Ser342 and Cys285, both residues critical for ligand recognition and partial agonism of PPARγ, may underlie the enhanced affinity of the glycosylated derivatives.^31,81^ The flexibility introduced by glycosidic substitution likely favors reversible, dynamic binding, a characteristic often associated with partial agonists that modulate receptor activity without full activation.^82^ Similar Pi-Cation contacts with Arg288, previously linked to improved structural stabilization in PPARγ ligands,^33^ further support this hypothesis. Together, these structural features suggest that glycosylation of RV derivatives may refine PPARγ interaction patterns, potentially contributing to neuroprotective modulation via antioxidant and anti-inflammatory signaling pathways.

The stronger binding affinities observed for the glycosylated derivatives toward the ERBB2 receptor highlight the potential role of glycosylation in modulating ligand–receptor recognition and structural stabilization. Meanwhile, an unfavorable acceptor-acceptor interaction was observed between polydatin and the Asp154 residue. Polydatin, in particular, displayed an interaction pattern dominated by hydrophobic and dispersion contacts, suggesting that apolar complementary surfaces may compensate for less favorable polar contacts such as the acceptor–acceptor geometry observed.^34^ This balance between the hydrogen-bonding capacity and hydrophobic packing could underline the increased conformational stability detected in the docking analysis. From a mechanistic perspective, these results imply that subtle structural modifications introduced by glycosylation can reshape the interaction network, refining the orientation and strength of binding to ERBB2.

The involvement of the ERBB2 receptor in PD pathogenesis is still largely unexplored. PD patient’s brains have shown decreased levels of ERBB in post-mortem analysis.^83^ ERBB2 primary functions in amplifying signals are mainly linked to MAPK and PI3K-Akt pathways, which modulate cell proliferation, differentiation, and survival, especially in glial cells. PI3K/Akt pathway activation can promote neural survival and decrease dopaminergic neurons apoptosis.^84^ Contrarily, inhibition of MAPK-related protein phosphorylation, especially p38 and JNK, has been associated with antiapoptotic and anti-inflammatory effects.^85^ Experimental research indicates that RV could exert neuroprotective effects by activating the PI3K/Akt pathway, thereby reducing neuronal death.^86–88^ Moreover, inhibition of the MAPK pathway by RV has been shown to increase the neuronal survival in rodent models of neurological diseases.^89^

Our results suggest that glycosylation plays a key role in modulating the binding affinity and interaction profiles of RV glycosylated derivatives with proteins involved in PD pathogenesis. The increased binding stability exhibited by polydatin and resveratrol-3-α-glucoside with the multiple target proteins analyzed and particularly with TNF-α and PPARγ, supports the hypothesis that additional hydrogen groups introduced through the glycosylation enhance hydrogen bonding and hydrophobic interactions. Moreover, glycosylation not only improves molecular interactions but also seems to enhance the stability and selectivity in ligand–receptor binding. Still, structural factors such as the glycosidic bond type and its steric effects appear to influence the ligand orientation and accessibility to active sites, potentially influencing receptor affinity. Polydatin exhibited the highest affinity for ERBB2 protein, a receptor with emerging implications in neurodegenerative processes, whose activation shows complex functional roles. These results provide an understanding of the molecular determinants underlying the mechanisms that may be involved in the bioactivity of RV and its glycosylated derivatives, yet experimental validation is necessary to fully elucidate their pharmacological potential in PD.

## 5 Conclusion

Considering these findings collectively, our data highlight the potential of glycosylated RV derivatives as promising candidates for targeting key molecular pathways associated with PD pathogenesis. The increased binding affinities observed for polydatin and resveratrol-3-α-glucoside suggest that structural optimization via glycosylation may improve the molecular stability and interaction profiles with multiple PD targets, especially with TNF-α, PPARγ, and ERBB2. These interactions align with already established neuroprotective mechanisms, including antioxidant, anti-inflammatory, and antiapoptotic effects. Despite these promising *in silico* results, further experimental studies are required to complete preclinical drug verification, assessing their pharmacokinetic properties, bioavailability, and therapeutic potential of these compounds in relevant biological models. Future studies should investigate whether these compounds can cross the BBB, their metabolic stability, and their efficacy in preclinical models of neurodegeneration. Understanding these aspects will be essential for translating these findings into clinical therapeutic applications for glycosylated RV derivatives in PD as well as other neurodegenerative disorders.

## Supplementary Material



## Notes

Table S1, grid box coordinates and dimensions used in molecular docking simulations; Table S2, molecular docking results of resveratrol, polydatin, and resveratrol-3-α-glucoside in key targets of Parkinson’s disease; Figure S1, binding interactions of resveratrol, polydatin, and resveratrol-3-α-glucoside within the EGFR receptor active site and resveratrol, polydatin, and resveratrol-3-α-glucoside molecular interactions; Figure S2, binding interactions of resveratrol, polydatin, and resveratrol-3-α-glucoside within the ALB receptor active site and resveratrol, polydatin, and resveratrol-3-α-glucoside molecular interactions; Figure S3, binding interactions of resveratrol, polydatin, and resveratrol-3-α-glucoside within the CASP3 receptor active site and resveratrol, polydatin, and resveratrol-3-α-glucoside molecular interactions; Figure S4, binding interactions of resveratrol, polydatin, and resveratrol-3-α-glucoside within the ESR1 receptor active site and resveratrol, polydatin, and resveratrol-3-α-glucoside molecular interactions; Figure S5, binding interactions of resveratrol, polydatin, and resveratrol-3-α-glucoside within the PTGS2 receptor active site and resveratrol, polydatin, and resveratrol-3-α-glucoside molecular interactions; Figure S6, binding interactions of resveratrol, polydatin, and resveratrol-3-α-glucoside within the SRC receptor active site and resveratrol, polydatin, and resveratrol-3-α-glucoside molecular interactions; Figure S7, binding interactions of resveratrol, polydatin, and resveratrol-3-α-glucoside within the ACE receptor active site and resveratrol, polydatin, and resveratrol-3-α-glucoside molecular interactions; Figure S8, binding interactions of resveratrol, polydatin, and resveratrol-3-α-glucoside within the MAP2K1 receptor active site and resveratrol, polydatin, and resveratrol-3-α-glucoside molecular interactions; Figure S9, TNF-α-cocrystallized ligand binding site and 3D and 2D representations showing interactions with key residues; Figure S10, PPARγ-cocrystallized ligand binding site and 3D and 2D representations showing interactions with key residues; Figure S11, ERBB2-cocrystallized ligand binding site and 3D and 2D representations showing interactions with key residues (PDF)

## Abbreviations Used

ADME: Absorption, Distribution, Metabolism and Excretion
Atad5: Atpase Family AAA Domain Containing Protein 5
BaSP: Bifidobacterium adolescentis
BBB: Blood–Brain Barrier
BP: Biological Process
CC: Cell Component
COX-2: cyclooxygenase-2
CTD: Comparative Toxicogenomics Database
CYP450: Cytochrome P450
ERBB2: Human Epidermal Growth Factor Receptor 2
GO: Gene Ontology
IL-6: Interleukin 6
KEGG: Kyoto Encyclopedia of Genes and Genomes
MF: Molecular Function
MMP: Mitochondrial Membrane Potential
NHorOH: Number H-bond Donors
NF-κB: Nuclear Factor Kappa-Light-Chain-Enhancer of Activated B Cells
NR: Nuclear Receptor
Nrf2: Nuclear Factor Erythroid 2-Related Factor 2
P-gp: P-glycoprotein
PDB: Protein Data Bank
PD: Parkinson’s Disease
PPARγ: Peroxisome Proliferator-Activated Receptor Gamma
PPI: Protein–Protein Interaction
RV: Resveratrol
RV-3-α-glucoside: resveratrol-3-α-glucoside
TNF-α: Tumor Necrosis Factor-Alpha
VD: Volume of Distribution
