# Supplementary material for: Glycosylation Matters: Network Pharmacology-Based and Molecular Docking Analysis of Resveratrol Glycosylated Derivatives on Parkinson’s Disease
Source: ACS Omega. 2026 Jan 23;11(5):7597–615. doi: 10.1021/acsomega.5c08887 (PMC12903038; doi:10.1021/acsomega.5c08887)
Supplement: Supplementary file 1 [file ao5c08887_si_001.pdf]

"Glycosylation matters: network pharmacology-based and molecular docking analysis of resveratrol glycosylated derivatives on Parkinson's disease"

Lucia E. Schimith[1,2]*, Elvis Martis[2], Stéphane Teletchea[2], Ana Muccillo-Baisch[1], Corinne Miral[2], Mariana A. Hort[1]

[1]Programa de Pós-graduação em Ciências da Saúde, Faculdade de Medicina, Universidade Federal do Rio Grande, 96203-900, Rio Grande, RS, Brasil;

[2]Nantes Université, CNRS, US2B, UMR 6286, 44322, Nantes, France.

Supporting Information

SMILES Codes

Compound	SMILES
Resveratrol	C1=CC(=CC=C1/C=C/C2=CC(=CC(=C2)O)O)O
Polydatin	C1=CC(=CC=C1/C=C/C2=CC(=CC(=C2)O[C@H]3[C@@H]([C@H]([C@@H]([C@H](O3)CO)O)O)O)O)O
Resveratrol-3-α-glucoside	OCC1OC(OC2=CC(O)=CC(\C=C\C3=CC=C(O)C=C3)=C2)C(O)C(O)C1O

Table S1. Grid box coordinates and dimensions used in molecular docking simulations.

		Grid box coordinates (Å)		
**Receptor**	**PDB ID**	**X**	**Y**	**Z**
TNF	7JRA	-14.378	-2.467	-26.322
EGFR	1M17	22.014	0.253	52.794
ALB	1H9Z	32.092	13.756	9.434
CASP3	1NMS	-9.183	-3.963	23.945
ESR1	1XPC	31.616	-1.861	24.613
PPARγ	1FM6	-1.722	69.521	35.872
PTGS2	5IKR	38.996	2.383	61.501
ERBB2	3RCD	13.415	2.748	27.535
SRC	8JN9	-17.822	75.904	106.807
ACE	1UZE	40.766	35.486	46.908
MAP2K1	3VVH	68.054	107.433	-32.097
**Box dimension**		20	20	20

Table S2. Molecular docking results of resveratrol, polydatin, and resveratrol-3-α-glucoside in key targets of Parkinson's disease.

		Binding energy (kcal/mol)		
**Receptor**	**PDB ID**	**Resveratrol**	**Polydatin**	**Resveratrol-3-α-glucoside**
TNF	7JRA	-8.6	-9.6	-9.3
EGFR	1M17	-7.5	-8.8	-8.6
ALB	1H9Z	-7.1	-8.9	-7.9
CASP3	1NMS	-6.4	-7.7	-7.6
ESR1	1XPC	-8.1	-8.6	-7.6
PPARγ	1FM6	-7.3	-8.9	-9.1
PTGS2	5IKR	-7.2	-6.5	-6.2
ERBB2	3RCD	-7.8	-9.4	-8.4
SRC	8JN9	-6.4	-7.8	-7.7
ACE	1UZE	-7.1	-8.9	-8.7
MAP2K1	3VVH	-8.0	-8.8	-8.7

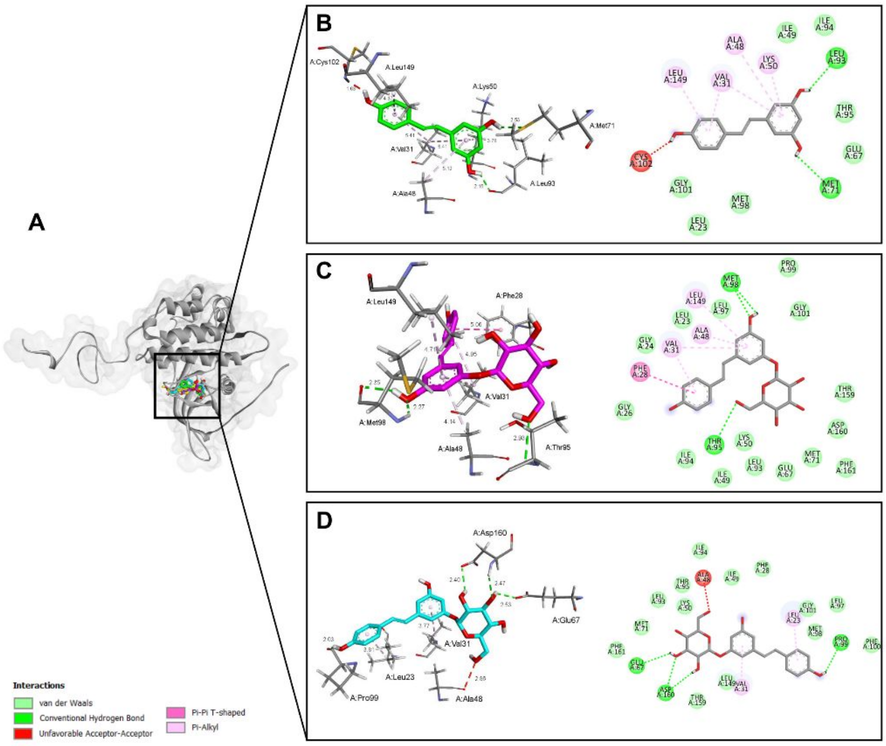

Figure S1. (A) Binding interactions of resveratrol, polydatin, and resveratrol-3-α-glucoside within the EGFR receptor active site. Resveratrol (B), polydatin (C), and resveratrol-3-α-glucoside (D) molecular interactions, with the panels on the left corresponding to the 3D structures and those on the right corresponding to the 2D diagrams. Letters before residue names indicate the corresponding protein chain in the PDB structure.

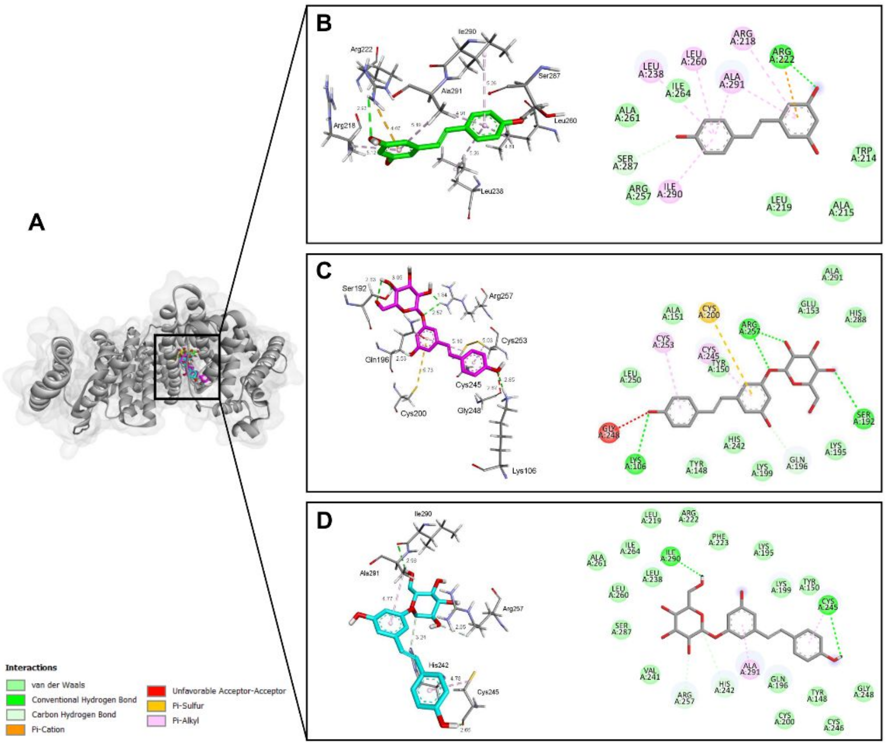

Figure S2. (A) Binding interactions of resveratrol, polydatin, and resveratrol-3-α-glucoside within the ALB receptor active site. Resveratrol (B), polydatin (C), and resveratrol-3-α-glucoside (D) molecular interactions, with the panels on the left corresponding to the 3D structures and those on the right corresponding to the 2D diagrams. Letters before residue names indicate the corresponding protein chain in the PDB structure.

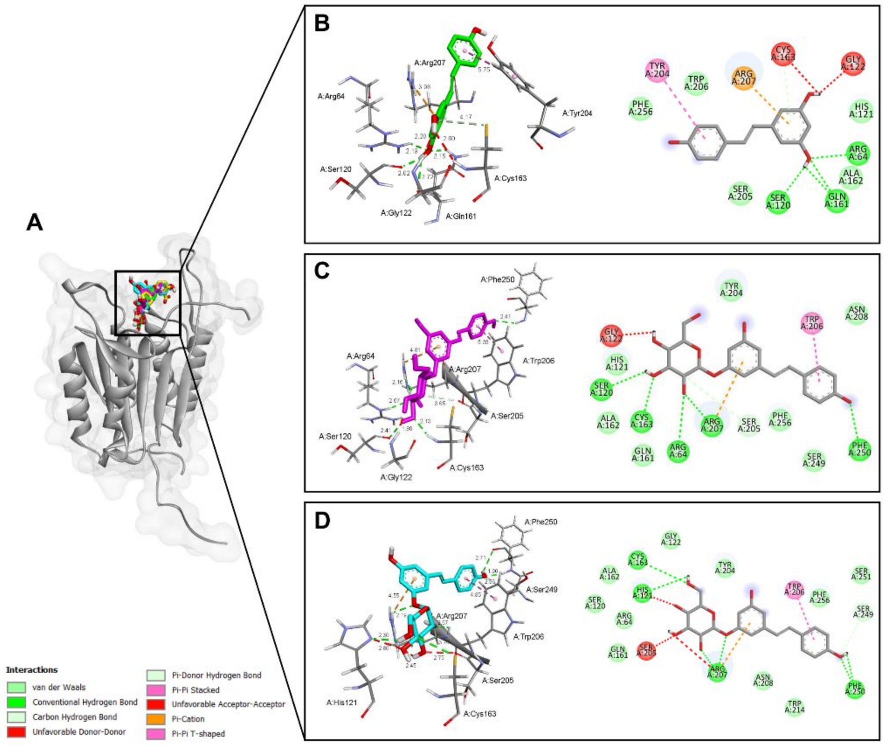

Figure S3. (A) Binding interactions of resveratrol, polydatin, and resveratrol-3-α-glucoside within the CASP3 receptor active site. Resveratrol (B), polydatin (C), and resveratrol-3-α-glucoside (D) molecular interactions, with the panels on the left corresponding to the 3D structures and those on the right corresponding to the 2D diagrams. Letters before residue names indicate the corresponding protein chain in the PDB structure.

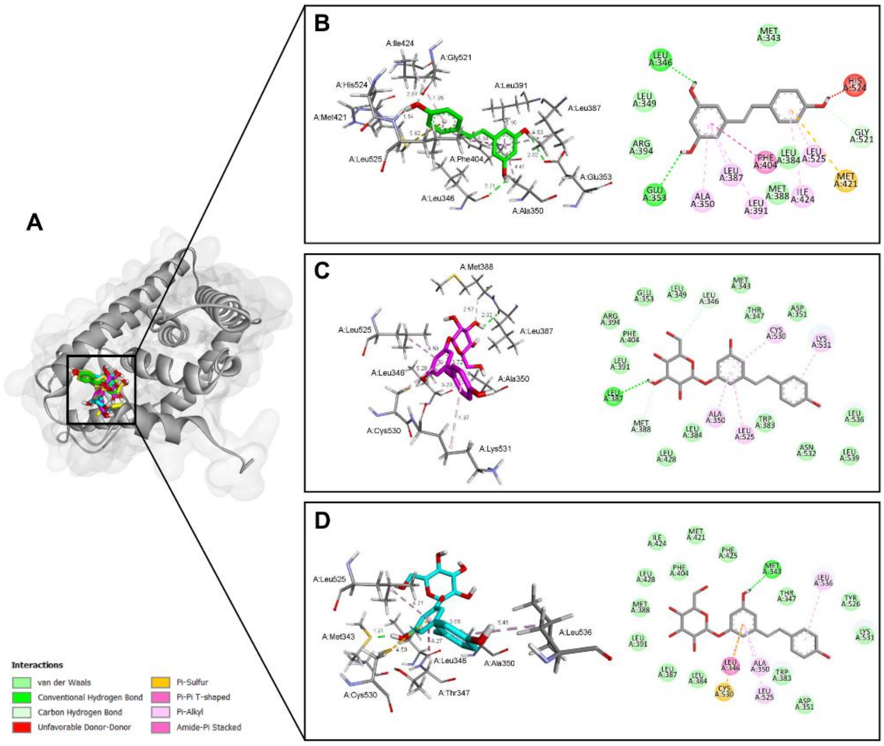

Figure S4. (A) Binding interactions of resveratrol, polydatin, and resveratrol-3-α-glucoside within the ESR1 receptor active site. Resveratrol (B), polydatin (C), and resveratrol-3-α-glucoside (D) molecular interactions, with the panels on the left corresponding to the 3D structures and those on the right corresponding to the 2D diagrams. Letters before residue names indicate the corresponding protein chain in the PDB structure.

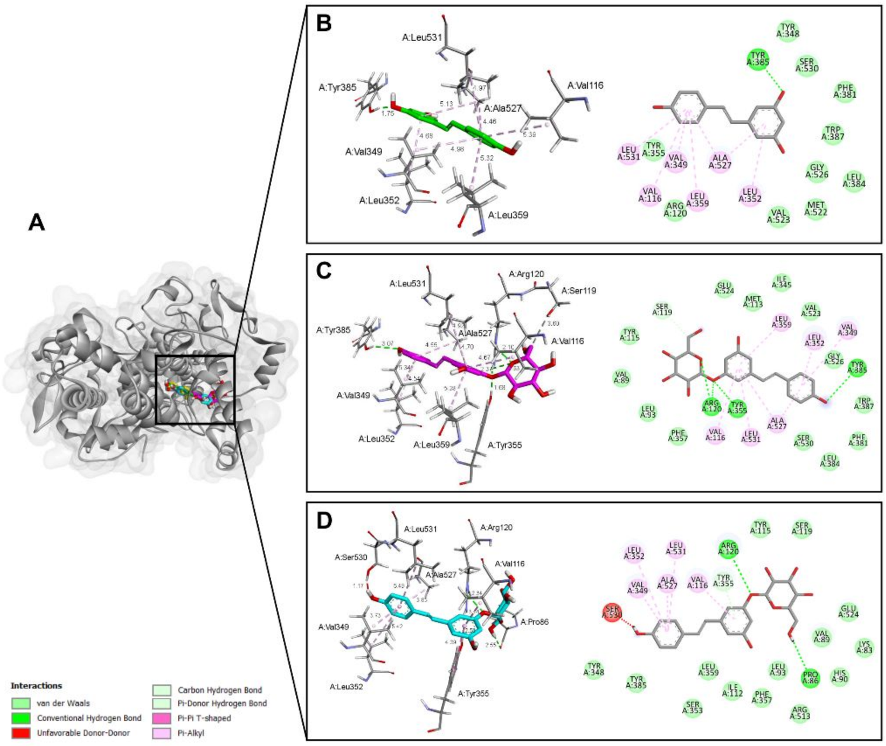

Figure S5. (A) Binding interactions of resveratrol, polydatin, and resveratrol-3-α-glucoside within the PTGS2 receptor active site. Resveratrol (B), polydatin (C), and resveratrol-3-α-glucoside (D) molecular interactions, with the panels on the left corresponding to the 3D structures and those on the right corresponding to the 2D diagrams. Letters before residue names indicate the corresponding protein chain in the PDB structure.

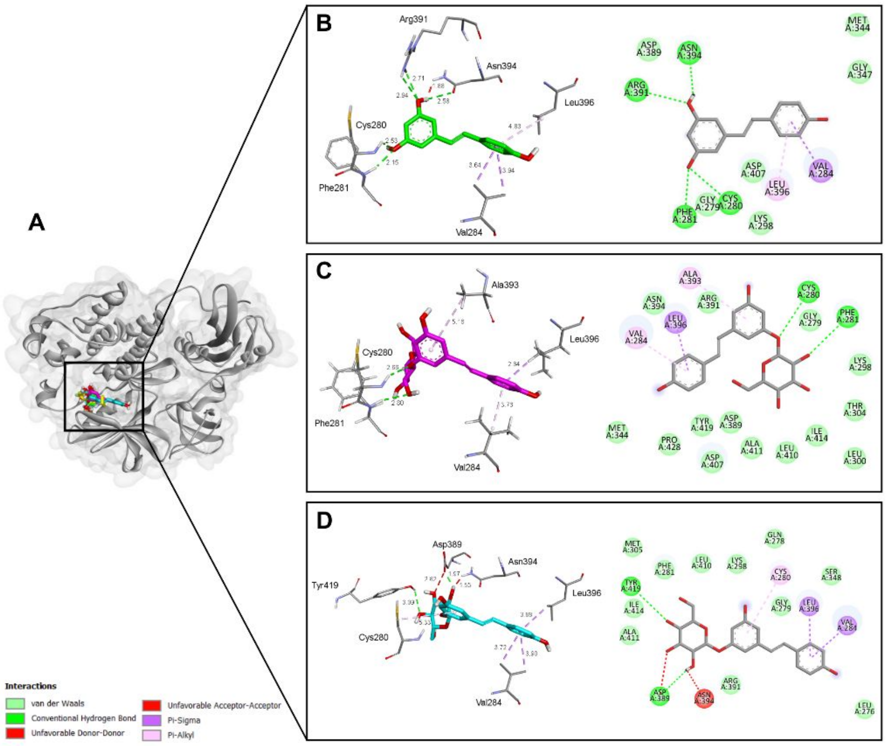

Figure S6. (A) Binding interactions of resveratrol, polydatin, and resveratrol-3-α-glucoside within the SRC receptor active site. Resveratrol (B), polydatin (C), and resveratrol-3-α-glucoside (D) molecular interactions, with the panels on the left corresponding to the 3D structures and those on the right corresponding to the 2D diagrams. Letters before residue names indicate the corresponding protein chain in the PDB structure.

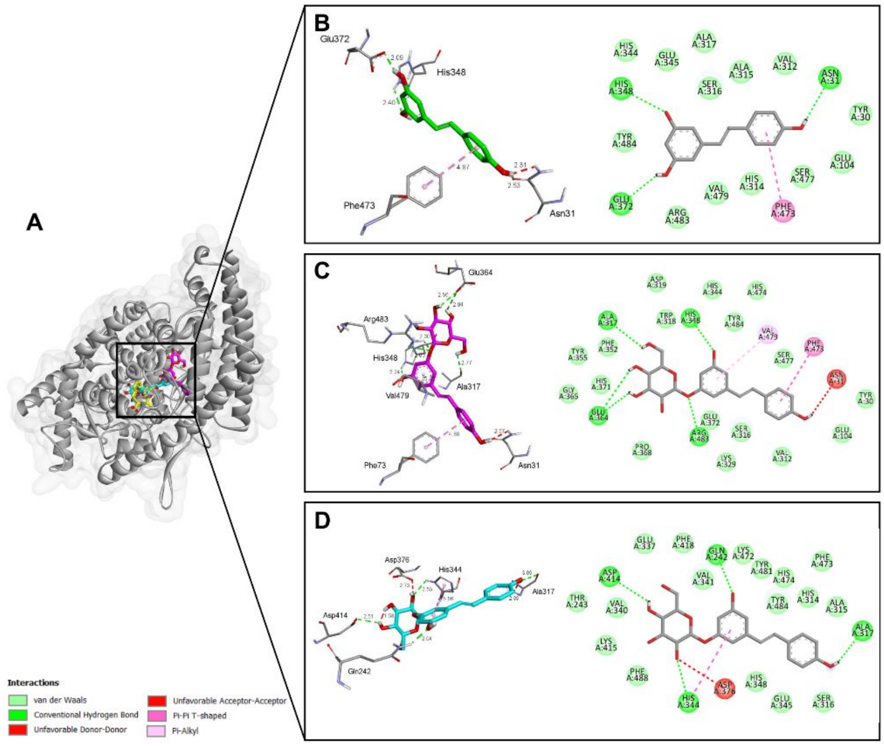

Figure S7. (A) Binding interactions of resveratrol, polydatin, and resveratrol-3-α-glucoside within the ACE receptor active site. Resveratrol (B), polydatin (C), and resveratrol-3-α-glucoside (D) molecular interactions, with the panels on the left corresponding to the 3D structures and those on the right corresponding to the 2D diagrams. Letters before residue names indicate the corresponding protein chain in the PDB structure.

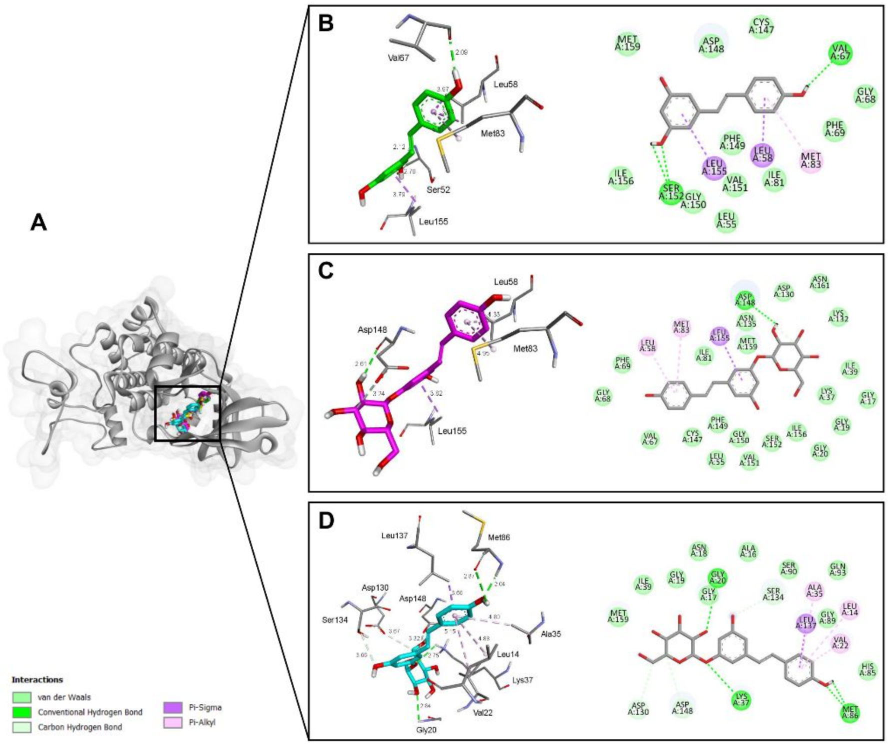

Figure S8. (A) Binding interactions of resveratrol, polydatin, and resveratrol-3-α-glucoside within the MAP2K1 receptor active site. Resveratrol (B), polydatin (C), and resveratrol-3-α-glucoside (D) molecular interactions, with the panels on the left corresponding to the 3D structures and those on the right corresponding to the 2D diagrams. Letters before residue names indicate the corresponding protein chain in the PDB structure.

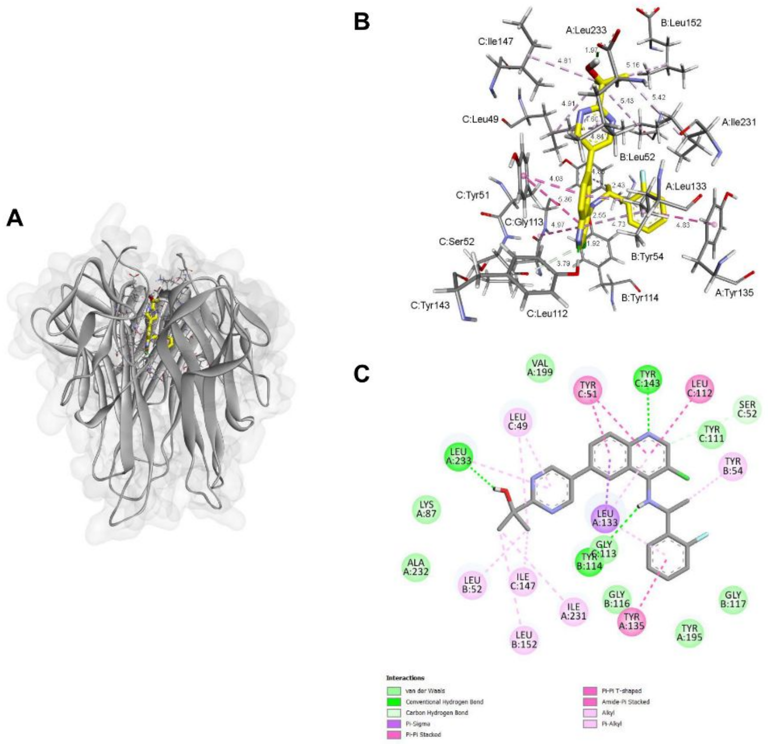

Figure S9. (A) TNF-α – co-crystallized ligand binding site; (B) 3D and (C) 2D representations showing interactions with key residues. Letters before residue names indicate the corresponding protein chain in the PDB structure.

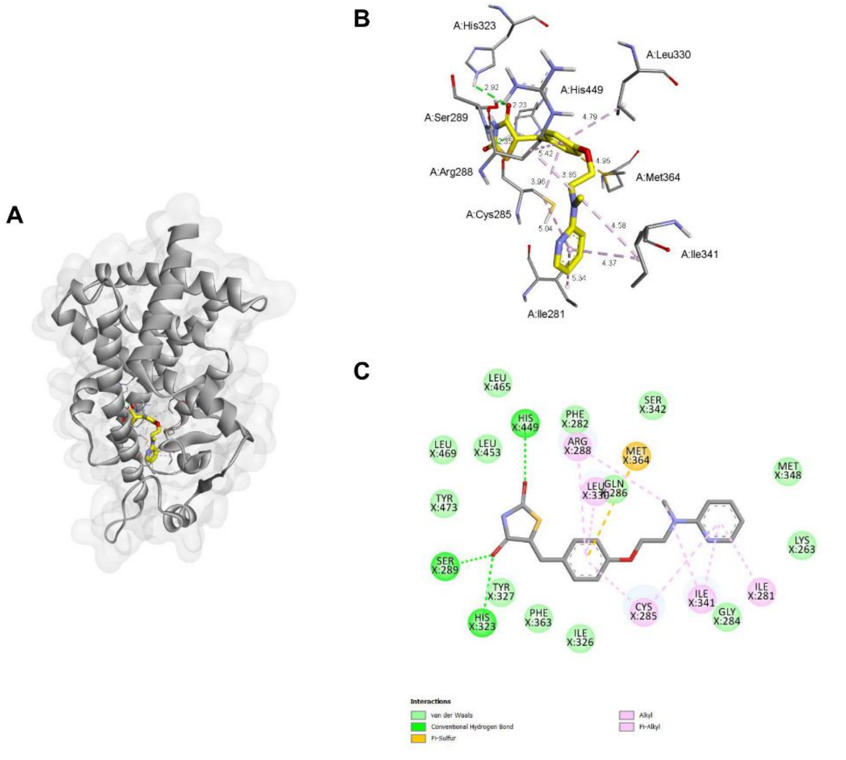

Figure S10. (A) PPARγ – co-crystallized ligand binding site; (B) 3D and (C) 2D representations showing interactions with key residues. Letters before residue names indicate the corresponding protein chain in the PDB structure.

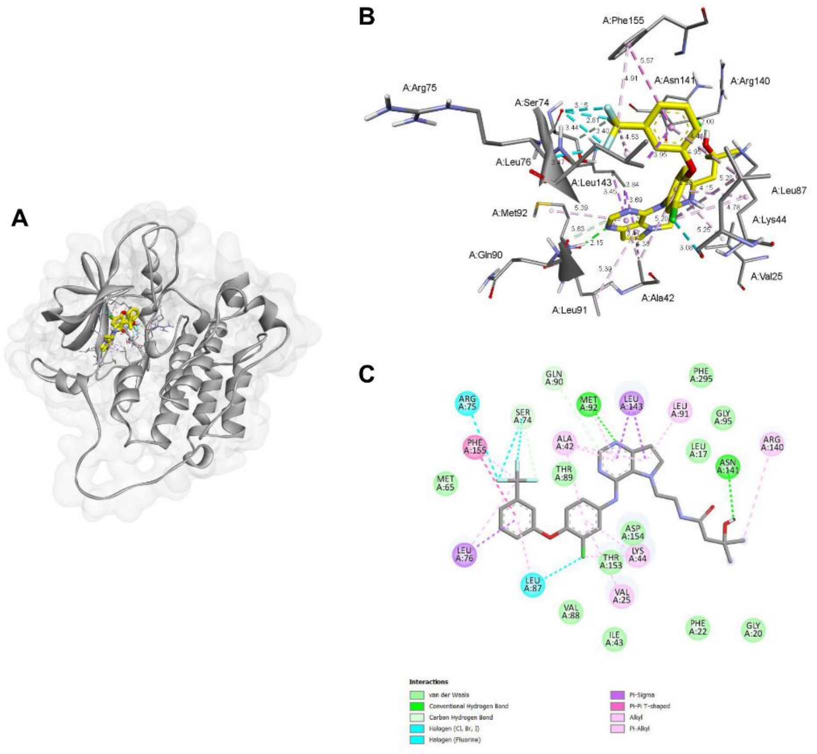

Figure S11. (A) ERBB2 – co-crystallized ligand binding site; (B) 3D and (C) 2D representations showing interactions with key residues. Letters before residue names indicate the corresponding protein chain in the PDB structure.
